# Interplay Between p53 and Wnt/β-Catenin Signaling in Colorectal Cancer: Associations with Mismatch Repair Status, Tumor Microenvironment, and Clinicopathological Outcomes

**DOI:** 10.3390/curroncol33030178

**Published:** 2026-03-21

**Authors:** Seiya Chiba, Shu Oikawa, Hiroyuki Mitomi, Yosuke Sasaki, Takahiro Hobo, Takuya Terunuma, Yumika Takano, Marin Hojo, Toshiko Yamochi, Noboru Yokoyama

**Affiliations:** 1 Department of Gastroenterological Surgery, Showa Medical University School of Medicine, Koto Toyosu Hospital, 5-1-38 Toyosu, Koto-ku 135-8577, Tokyo, Japan; seiyachiba@med.showa-u.ac.jp (S.C.); takahiroho@med.showa-u.ac.jp (T.H.); takuya2145@med.showa-u.ac.jp (T.T.); gm22-y022@med.showa-u.ac.jp (Y.T.); marin.h.20@med.showa-u.ac.jp (M.H.); noboru.y@med.showa-u.ac.jp (N.Y.); 2 Department of Diagnostic Pathology, Showa Medical University School of Medicine, 1-5-8 Hatanodai, Shinagawa-ku 142-8555, Tokyo, Japan; hmitomi@med.showa-u.ac.jp (H.M.); sasaki0224@med.showa-u.ac.jp (Y.S.); onizuka@med.showa-u.ac.jp (T.Y.); 3 Division of Gastroenterology, Department of Medicine, Showa Medical University School of Medicine, 1-5-8 Hatanodai, Shinagawa-ku 142-8555, Tokyo, Japan

**Keywords:** colorectal cancer, p53, Wnt/β-catenin signaling, mismatch repair, tumor budding, poorly differentiated cluster, image analysis, prognosis

## Abstract

The interplay between TP53 alterations and Wnt/β-catenin signaling in colorectal cancer (CRC) remains unclear regarding mismatch repair (MMR) status, tumor budding (TB), poorly differentiated cluster (PDC), and prognosis. We analyzed 146 resected CRC cases, quantifying p53, Wnt3, and β-CTN indices and assessing MMR by PMS2 and MSH6 immunohistochemistry. p53 overexpression was associated with younger patients, left-sided tumors, nodal metastasis, and advanced stage, whereas wild-type tumors showed more mucinous differentiation. Deficient MMR was enriched among wild-type p53 cases. Principal component analysis identified distinct axes defined by p53, Wnt3, and β-CTN. Despite comparable Wnt3 levels, nuclear β-CTN accumulation was enhanced in tumors with aberrant (overexpression or null) p53 tumors, with increased TB and PDC indices. Low nuclear β-CTN independently predicted recurrence in stage I–III disease and worse overall survival in proficient MMR tumors (HR 3.07 and 2.52; *p* = 0.03 for both). A composite score integrating p53 binary status (aberrant vs. wild) with Wnt3 and whole β-CTN indices predicted survival beyond stage; each 1-point increase conferred a 2.56- and 1.77-fold higher risk of cancer-specific and overall mortality (*p* = 0.004 and 0.04). These findings suggest that p53 dysfunction is associated with alterations in Wnt/β-CTN signaling and that integrating signaling markers with staging may improve prognostic assessment in colorectal cancer.

## Simple Summary

Colorectal cancer is biologically diverse, and treatment decisions are largely guided by tumor stage. In this study, we examined how abnormalities in p53, a key tumor suppressor protein frequently altered in cancer, are associated with alterations in the Wnt/β-catenin signaling pathway, including its activating ligand Wnt3, which regulates cell growth and invasion. β-catenin normally functions as a cell adhesion molecule at the cell membrane, but when Wnt signaling becomes dysregulated, it moves to the nucleus, where it activates genes that promote tumor progression. We also assessed how these alterations relate to DNA repair status and invasive-front features such as tumor budding and poorly differentiated clusters. Tumors with abnormal p53 expression showed increased nuclear β-catenin accumulation and more active invasive fronts, with higher tumor budding and poorly differentiated cluster formation, despite similar levels of Wnt3 expression. In contrast, tumors with intact p53 were more often associated with defects in DNA repair mechanisms. Although the pathological stage remained the strongest determinant of survival, combining key tumor markers with standard clinical assessment provided additional prognostic information and may help identify patients at higher risk of recurrence.

## 1. Introduction

Colorectal cancer (CRC) is a biologically heterogeneous disease characterized by progressive acquisition of driver alterations in APC, KRAS, TP53, and the Wnt/β-catenin pathway together with epigenetic dysregulation, shaping tumor behavior, clinical outcome, and consensus molecular subtypes (CMSs) [1]. These trajectories diverge into chromosomal instability tumors and microsatellite instability (MSI) tumors arising from mismatch repair (MMR) deficiency, each with distinct biological and clinical features [1]. TP53 disruption is among the most prevalent events in colorectal carcinogenesis, reported in roughly one-half or more of CRCs [2,3,4]. Most TP53 mutations are missense alterations [4,5] that disrupt wild-type tumor suppressor function and may also confer gain-of-function properties, promoting tumor progression through aberrant transcriptional programs [6,7]. These variants often stabilize the p53 protein, producing an overexpression pattern on immunohistochemistry (IHC) widely used as a surrogate for TP53 mutation status [2,4,5]. Although extensively investigated as a prognostic biomarker, p53 remains clinically controversial in CRC [5,8,9,10,11,12,13,14], possibly due to differences in scoring systems, cutoff selection, classification of null versus overexpression patterns, and limited integration with molecular subtypes.

The MSI pathway, resulting from inactivation of mismatch repair (MMR) genes such as MLH1, MSH2, MSH6, and PMS2, characterizes a hypermutated and immune-rich subset of CRC that largely overlaps with the CMS1 molecular subtype [1]. Immunohistochemical analyses of p53 in CRC have explored its distribution across microsatellite-stable (MSS) and MSI-high tumors, with recently refined phenotypic classifications of p53 expression patterns such as wild-type, overexpression, complete loss, and restricted patterns examined in relation to MMR status and TP53 mutational background [2,13]. Notably, MSI-high tumors are overrepresented in mucinous CRC [15,16], underscoring the association between molecular subtype and histopathological phenotype. However, whether these emerging p53 expression patterns are similarly linked to mucinous differentiation has not been clearly established.

The Wnt/β-catenin signaling cascade, driven by nuclear β-catenin (β-CTN), is a central driver of colorectal carcinogenesis, regulating proliferation, invasion, inflammation, and epithelial–mesenchymal transition (EMT) within the tumor microenvironment [17]. Both Wnt/β-CTN activation and TP53 dysfunction have been implicated in EMT-related invasive phenotypes in experimental models of colorectal cancer progression [18]. Mutant p53 has been shown to influence canonical Wnt/β-CTN signaling through the PI3K/AKT pathway and Ser552 phosphorylation of β-CTN in colon cancer cells [19]. In addition, gain-of-function TP53 mutations, particularly in the setting of wild-type allele loss, have been investigated in relation to inflammatory transcriptional programs and invasive behavior [6]. Increasing evidence also points to possible reciprocal interactions between TP53 and Wnt/β-CTN signaling, as suggested by studies examining wild-type and mutant p53 in canonical Wnt regulation [7,19]. Together, these lines of evidence highlight the potential influence of TP53 status on Wnt/β-CTN-driven tumor biology.

Wnt3 and Wnt3a are upregulated in CRCs and sustain β-CTN-dependent transcriptional activity, contributing to proliferation, invasion, and EMT-related programs [20,21]. Colon cancer models have revealed that interference with Wnt ligand secretion, including Wnt3, disrupts canonical Wnt activity and compromises tumor-forming capacity [22]. In addition, intracellular signaling pathways, including PI3K-mediated signaling, have been explored as regulators of β-CTN stabilization and transcriptional activity [23,24]. Clinically, the prognostic impact of Wnt/β-CTN signaling remains heterogeneous. While Wnt3a expression has been linked to adverse outcomes in a CRC cohort [20], independent studies have yielded variable findings [25,26]. Similarly, the prognostic relevance of β-CTN expression appears to vary according to its subcellular localization, particularly nuclear versus membranous patterns [12,25,26,27,28,29].

Histologically, tumor budding (TB) and poorly differentiated clusters (PDCs) are considered morphological correlates of EMT [30,31] and robust indicators of aggressive tumor behavior in CRC [32,33,34,35]. At the invasive front, TB areas frequently demonstrate nuclear β-CTN localization together with increased expression of EMT-related factors, including Slug [36]. This region is also characterized by lymphocytic infiltration, including CD8^+^ T-cell subsets, and dynamic stromal interactions that influence tumor behavior [30,34]. In line with the inflammatory milieu at the invasive front, TP53 gain-of-function mutation combined with loss of wild-type TP53 drives the activation of inflammatory transcriptional programs that are coupled to MAPK signaling [18]. Clinically, TP53 alterations are associated with reduced cytotoxic lymphocyte density [3,37], although overall T-cell infiltration does not consistently differ by TP53 status across molecular subtypes [38].

Despite advances in molecular classification, the biological and clinical significance of the interplay between TP53 status and Wnt/β-CTN signaling in CRC remains incompletely understood with respect to MMR status, tumor microenvironment, and invasive-front morphology. Therefore, we aimed to comprehensively evaluate the interaction between p53 expression patterns and image-based quantification of Wnt/β-CTN signaling and to examine their associations with invasive-front histomorphological features, including TB and PDC, and clinical outcomes in a well-characterized CRC cohort.

## 2. Materials and Methods

### 2.1. Patients and Materials

This retrospective study was conducted in accordance with the Declaration of Helsinki (2013 revision) and approved by the Institutional Review Board of the Showa Medical University Ethics Committee (No. 2025-0335). General consent for research use of clinical data and surgical specimens is routinely obtained at our institution. The requirement for study-specific informed consent was waived due to the retrospective nature of this study.

A total of 146 patients with CRC who underwent primary resection at Showa Medical University Koto Toyosu Hospital (2017–2019) were retrospectively reviewed. Patient selection and exclusion are shown in Figure S1. The cohort included 88 men (60.3%) and 58 women (39.7%) with a mean age of 74.1 years. Adjuvant chemotherapy was administered to 54 patients (37.0%), and 15 (10.3%) received palliative chemotherapy as salvage treatment. During follow-up, 24 patients (16.4%) developed recurrence and 31 (21.2%) died, including 22 (15.1%) cancer-related deaths, with a median follow-up of 2155 days (approximately 5.9 years) among survivors (*n* = 115). Of 17 stage IV cases, KRAS and BRAF mutations, MSI status, and uridine diphosphate glucuronosyltransferase 1A1 (UGT1A1) data were available for 16. The details are shown in Supplementary Tables S1 and S2.

### 2.2. Histological Evaluation

Resected specimens were fixed in 10% neutral-buffered formalin, sliced at 5 mm, and paraffin-embedded. Hematoxylin and eosin (H&E)-stained 4 µm sections were reviewed independently by two investigators (S.C. and H.M., the latter a board-certified pathologist) blinded to clinical data. Pathological staging followed the UICC TNM 8th edition [39]. Tumor laterality was categorized as right- (proximal to the splenic flexure) or left-sided. Representative H&E sections were digitized at ×40 using the NanoZoomer S60 (Hamamatsu Photonics, Hamamatsu City, Japan) to generate a whole slide image (WSI), and the same block was used for IHC.

### 2.3. Definition and Image-Based Evaluation of TB and PDC

TB was defined as a single cell or a cluster of ≤ 4 tumor cells according to the ITBCC 2016 recommendations [33], and PDC was defined as solid clusters of 5–10 tumor cells lacking glandular differentiation, following modified definitions from previous studies [35]. Image-based quantification of TB and PDC was performed using ImageJ/Fiji (ImageJ, version 1.54; National Institutes of Health, Bethesda, MD, USA) (National Institutes of Health, Bethesda, MD, USA; https://fiji.sc/). For each case, five representative high-power field images (×40 objective) at the invasive front were captured from H&E WSIs, saved as JPEG files, analyzed to derive four quantitative indices (TB count, TB area, PDC count, and PDC area), and calculated as cumulative measurements across the five fields. Detailed procedures are provided in Section 1 of Supplementary File S1. 

### 2.4. Histological Scoring of Inflammation and Desmoplasia

Inflammatory cell infiltration and desmoplastic reaction were evaluated using the same H&E images used for TB and PDC assessment based on five representative high-power fields at the invasive front, and they were semi-quantitatively graded according to the proportion of stromal involvement within each field. Inflammation and desmoplasia were scored as 0 (absent), 1 (<50% involvement of the tumor–stroma interface), or 2 (≥50% involvement). The final inflammation and desmoplasia scores were calculated as the sum of the scores across the five evaluated high-power fields. Inflammation was further classified by predominant inflammatory cell type (lymphoplasmacytic, neutrophilic, eosinophilic, or histiocytic), and desmoplasia was categorized as either sclerotic or florid. Representative images with corresponding scores and classifications are shown in Supplementary Figure S2.

### 2.5. Antibodies and Staining Procedures

From each representative block, serial 4 μm sections were prepared for IHC. After heat-induced epitope retrieval (BOND Epitope Retrieval Solution 1 or 2; Leica Biosystems, Newcastle, UK), slides were stained on an automated platform (BOND-III; Leica Biosystems) using antibodies against p53 (mouse monoclonal, clone DO-7, prediluted; Leica Biosystems), β-catenin (mouse monoclonal, clone 17C2, prediluted; Leica Biosystems), Wnt3 (rabbit polyclonal, 1:400, LS-C175701; LSBio, Seattle, WA, USA), PMS2 (rabbit monoclonal, clone EPR3947, 1:200; Abcam, Cambridge, UK), and MSH6 (rabbit monoclonal, clone EPR3945, 1:500; Abcam). Detection was performed using a horseradish peroxidase-labeled polymer system (BOND Compact Polymer; Leica Biosystems), with 3,3′-diaminobenzidine as chromogen and methyl green or hematoxylin counterstaining. WSIs of all IHC slides were acquired at ×40 magnification using a NanoZoomer scanner.

### 2.6. Quantitative Analysis of p53, Wnt3, and β-CTN Immunostainings

Representative tumor fields were selected from p53-stained WSIs, and tumor cell nuclei were manually counted using the Cell Counter plugin in ImageJ/Fiji. Multiple high-power field images were analyzed until more than 1000 tumor nuclei were evaluated per case to calculate the p53 labeling index (%) in Section 2 of Supplementary File S1. Based on established criteria [4], p53 expression was categorized into four patterns: overexpression (strong diffuse nuclear staining in ≥80% of tumor cells), null (absence of nuclear staining), cytoplasmic (predominant cytoplasmic staining), and wild-type (weak to moderate nuclear staining in <80% of tumor cells, with or without cytoplasmic staining).

Wnt3-positive areas were quantified using a threshold-based WSI analysis approach. The tumor-specific upper threshold was set at the minimum intensity that largely excluded non-neoplastic signals while preserving tumor-specific staining. The entire-tumor upper threshold, which encompassed the whole tumor epithelium while minimizing stromal staining, was adopted as the higher upper threshold. In practical terms, the tumor-specific upper threshold captured signals stronger than those in non-neoplastic epithelium, whereas the entire-tumor upper threshold included both tumor and non-neoplastic epithelial staining while excluding stromal signals. The Wnt3 index (%) was calculated as the proportion of immunoreactive tumor epithelium defined by the tumor-specific threshold relative to the whole tumor area defined by the higher upper threshold in Section 3 of Supplementary File S1.

Whole and nuclear β-CTN indices were assessed in representative invasive-front fields from β-CTN-stained WSIs, where nuclear accumulation is most prominent [12,27,28,36]. The whole β-CTN index (%) was quantified using a threshold-based image analysis approach, in the same manner as for the Wnt3 index. The nuclear β-CTN index (/cm^2^) was defined as the number of β-CTN-positive tumor nuclei within the tumor area defined by the higher upper threshold in Section 4 of Supplementary File S1.

### 2.7. PMS2 and MSH6 Immunostaining Assessment

IHC for PMS2 and MSH6 was performed to determine the mismatch repair (MMR) status. Nuclear staining in tumor cells was evaluated in comparison with adjacent non-neoplastic mucosa and lymphocytes as internal positive controls. Complete loss of nuclear expression of either protein was interpreted as MMR deficiency (dMMR), whereas preserved nuclear staining indicated MMR proficiency (pMMR). This two-antibody approach has been proposed as a practical surrogate for the conventional four-marker panel (MLH1, PMS2, MSH2, and MSH6) in CRC. PMS2 and MSH6 were selected because they represent the key heterodimer partners of MLH1 and MSH2, respectively, and their loss reliably reflects inactivation of the corresponding MMR pathways [40,41]. Additional MLH1 and MSH2 IHC or MSI testing was not systematically performed in this study.

### 2.8. Statistical Analysis

Group differences and correlations were analyzed using the χ^2^ or Fisher’s exact test, the Mann–Whitney *U* test, and Spearman’s rank coefficient. Hierarchical clustering (Ward.D2, Euclidean distance) was applied to standardized IHC variables (p53 labeling, whole and nuclear β-CTN indices, and Wnt3 expression), which were standardized using z-score transformation (mean = 0, SD = 1) prior to clustering. The optimal number of clusters was determined based on dendrogram and silhouette analyses, and k-means clustering was used for validation based on dendrogram and silhouette analyses. Overall (OS), cancer-specific (CSS), and recurrence-free survival (RFS) were defined from surgery to death from any cause, death from CRC, or recurrence/death from CRC, respectively. Survival analyses were performed using the Kaplan–Meier method with the log-rank test. Optimal cutoff values for continuous variables were determined independently for each analysis cohort using the maximally selected log-rank statistic in an exploratory manner, and hazard ratios (HRs) and 95% confidence intervals (CIs) were estimated by Cox proportional hazards models. Time-dependent receiver operating characteristic (ROC) analysis was performed, and the area under the curve (AUC) was calculated to evaluate prognostic discrimination. The composite score was evaluated for incremental prognostic value using nested Cox models with likelihood-ratio tests. Interobserver agreement was evaluated using Cohen’s κ statistic. All analyses were two-sided, with *p* < 0.05 considered significant, and they were conducted in R version 4.3.1 (R Foundation, Vienna, Austria) using RStudio 2023.12.1.

## 3. Results

### 3.1. Clinicopathological Characteristics

For the overall cohort, the mean tumor size was 44.6 mm. Most tumors were well or moderately differentiated adenocarcinomas (143 cases, 98%), with only three cases (2%) showing poor differentiation; all poorly differentiated tumors were of the solid type according to the Japanese Classification [42]. A mucinous component was present in 31 cases (21.2%). Nearly half of the tumors were classified as pT3 (72 cases, 49.3%), and nodal metastasis was present in 57 patients (39.0%). Pathological stages I, II, III, and IV were observed in 28 (19.2%), 59 (40.4%), 42 (28.8%), and 17 (11.6%) cases, respectively (Supplementary Table S3).

Compared with stage I–III cases, patients with stage IV disease had larger tumors (median size, 65 vs. 40 mm) and showed a higher frequency of moderately differentiated adenocarcinoma (76.5% vs. 45.7%), as well as more advanced pT (pT4, 41.2% vs. 18.6%) and pN (pN1 + 2, 58.9% vs. 32.6%) categories. Among the tested stage IV cases, KRAS mutations (62.5%) and UGT1A1 heterozygous variants (43.8%) were detected, with no BRAF mutations or MSI-high tumors. Adjuvant and salvage chemotherapies were administered to 41.1% of stage I–III and 88.2% of stage IV patients, respectively. Among 15 patients with stage IV disease, eight (53.3%) received two or more lines of salvage chemotherapy (Supplementary Table S4).

### 3.2. Assessment of TB/PDC and Inflammation/Desmoplasia

The TB count ranged from 0 to 33 (median, 6 per case). The area of individual TBs ranged from 37 to 499 μm^2^ (median, 211 μm^2^), and the cumulative TB area showed a wide distribution (70–8642 μm^2^; median, 1641 μm^2^). For PDC, the count ranged from 0 to 23 (median, 4), with individual areas of 337–1982 μm^2^ (median, 895 μm^2^) and cumulative areas of 547–20,294 μm^2^ (median, 4664 μm^2^).

The inflammation score ranged from 0 to 13 (median, 4.0), and the desmoplasia score ranged from 1 to 10 (median, 5.0). Lymphoplasmacytic infiltration was the predominant inflammatory pattern (81 cases, 55.5%), followed by histiocytic (25, 17.1%), neutrophilic (20, 13.7%), and eosinophilic (seven, 4.8%) infiltration, while no inflammatory cell infiltration was identified in 13 cases (8.9%). Weighted κ analysis indicated almost perfect interobserver agreement for inflammation and desmoplasia (κ = 0.838, 0.857, respectively; both *p* < 0.001). Representative histological images are shown in Supplementary Figure S2.

### 3.3. Immunohistochemical Findings

For p53, faint nuclear staining in adjacent non-neoplastic crypt epithelium and stromal cells served as an internal positive control. p53 expression was classified as overexpression in 73 cases (50.0%), null in 34 (23.3%), and wild-type in 39 (26.7%), with aberrant expression (overexpression or null) in 107 cases (73.3%); image analysis of a median of 1206 (range, 1009–2082) tumor nuclei per case showed a median p53 positivity of 96% (range, 81–99%), 0%(range, 0–0%), and 17% (range, 5–40%) in the respective groups. Although cytoplasmic staining was included in the predefined classification scheme, no cases with a cytoplasmic pattern were observed in this cohort. In tumors with p53 overexpression, nuclear staining was typically diffuse and uniform throughout the tumor, including the invasive front, whereas wild-type patterns showed scattered nuclear staining without specific enrichment at the invasive front.

Wnt3 staining was predominantly observed along the cytoplasmic basal aspect of both non-neoplastic crypt epithelial cells and tumor cells, showing relatively uniform cytoplasmic labeling within the tumor epithelium. In some tumors, staining intensity was stronger in superficial regions and gradually decreased toward deeper areas. Quantitative image analysis demonstrated variable cytoplasmic expression across the cohort, with a median Wnt3-positive area of 32.7% (range, 0.5–88.1%).

β-CTN showed distinct membranous staining in crypt epithelial cells in non-neoplastic mucosa, whereas in CRC, it was predominantly membranous with variable cytoplasmic and focal nuclear localization, with occasional loss of membranous staining and cytoplasmic and/or nuclear accumulation in TB at the invasive front. The median whole β-CTN index was 2.9% (range, 0.3–19.1%), and the median nuclear β-CTN index was 5.3/cm^2^ (range, 0–45.9). The whole and nuclear indices showed a strong positive correlation (Spearman’s *ρ* = 0.793, *p* < 0.001; Supplementary Figure S3).

PMS2 and MSH6 IHCs revealed preserved nuclear expression in the majority of cases. Loss of nuclear staining was identified in 14 cases (9.6%) for PMS2 and in one case (0.7%) for MSH6, resulting in 15 tumors (10.3%) classified as dMMR, with the remaining 131 cases (89.7%) as pMMR. Interobserver concordance between the two investigators (S.C. and H.M.) was excellent (Cohen’s κ = 0.986, *p* < 0.001).

**Figure 1:**
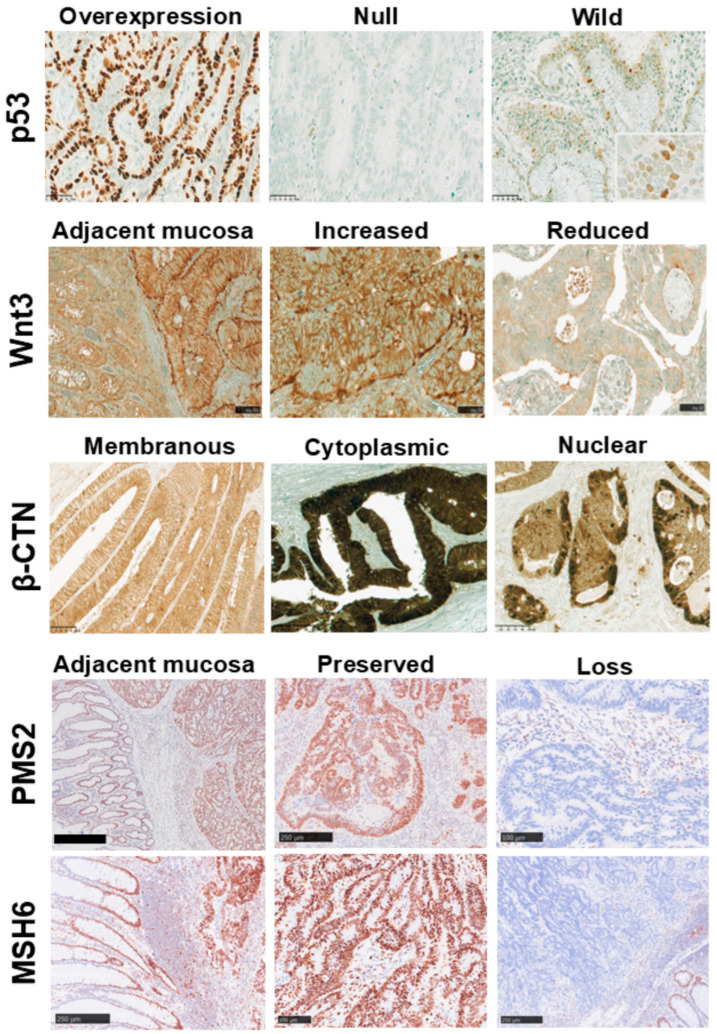
Representative IHC patterns of p53, Wnt3, β-CTN, PMS2, and MSH6. **p53:** Overexpression, null, and wild-type patterns are shown, exhibiting diffuse strong, absent, and weak to moderate heterogeneous nuclear staining, respectively. An inset in the wild-type panel highlights the characteristic nuclear staining pattern (scale bar, 50 μm). **Wnt3:** Adjacent mucosa (**left half**) shows weak basal cytoplasmic staining, whereas stronger staining is observed in the tumor area at the tumor–non-neoplastic interface (**right half**). Increased and diffuse cytoplasmic labeling and reduced staining intensity in low-expression tumor areas are also demonstrated (scale bars, 100 μm). **β-CTN:** Membranous staining with distinct cell borders, densely distributed cytoplasmic staining, and focal nuclear localization are shown (scale bars, 100 μm). **PMS2 and MSH6:** Adjacent mucosa (**left half**) and tumor areas (**right half**) show preserved nuclear staining (scale bars: PMS2, 500 μm; MSH6, 250 μm), including preserved expression in the deeper part of the tumor (scale bars: 250 μm and 100 μm). Complete loss of nuclear staining is observed in the tumor (scale bars: 100 μm and 250 μm).

Representative IHC images for each marker are presented in Figure 1.

### 3.4. Clinicopathological Features Stratified by Three-Tier and Binary p53 IHC Classifications

Table 1 summarizes clinicopathological features by p53 IHC classification. In the three-tier classification, p53 status was significantly associated with age, tumor location, lymph-node status, and pathological stage (all *p* ≤ 0.013). Pairwise analyses showed that p53 overexpression was more frequent in younger patients, left-sided tumors, node-positive cases, and advanced-stage disease than in the wild-type group (all adjusted *p* ≤ 0.019; Supplementary Figure S4), and in the binary model, aberrant p53 expression was additionally associated with non-mucinous histology and smaller tumor size (all *p* ≤ 0.040).

### 3.5. Comparison of TB/PDC Indices and Distribution Patterns of Inflammation/Desmoplasia Among Three-Tier and Binary p53 IHC Classifications

**Figure 2:**
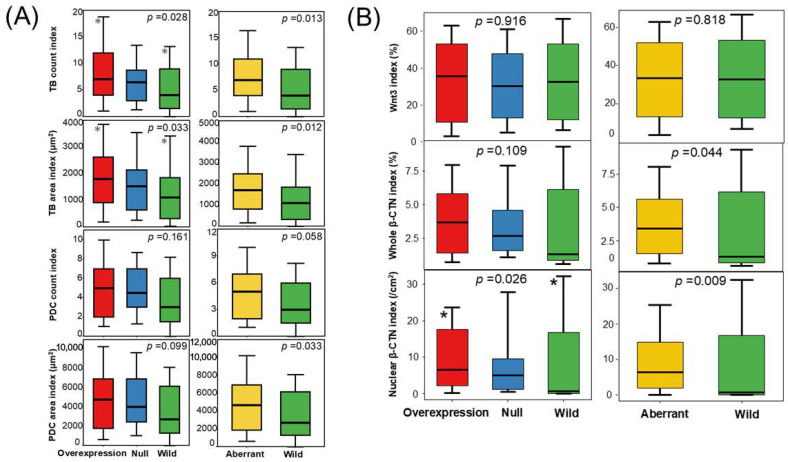
Associations of Wnt3, β-CTN, TB, and PDC with p53 status. (**A**) Distribution of TB and PDC indices across three-tier (**left**) and binary (**right**) p53 IHC classifications. Two pairs of asterisks denote significant differences in TB counts and TB area indices between the overexpression and wild-type groups (Holm-adjusted *p* = 0.027 and 0.036, respectively). (**B**) Box plots comparing Wnt3 and β-CTN indices according to the three-tier (**left**) and binary (**right**) p53 IHC classifications. A pair of asterisks indicates a significant difference between the overexpression and wild-type groups (adjusted *p* = 0.038). In panels (**A**,**B**), box plots show the median and interquartile range (25th–75th percentiles), with whiskers indicating the 10th and 90th percentiles.

TB count and area indices were significantly higher in the p53 overexpression group than in the wild-type group (adjusted *p* = 0.027 and 0.036, respectively) and remained elevated in the aberrant group under binary classification (*p* = 0.013 and *p* = 0.012, respectively). The PDC area index was also higher in the aberrant group (*p* = 0.033; Figure 2A and Supplementary Table S5).

No significant associations were observed for inflammation scores or subtypes or for desmoplasia scores or patterns. Across all groups, lymphoplasmacytic inflammation was the most frequent subtype (approximately 50–65%), and desmoplasia was distributed almost evenly between the two patterns (Supplementary Table S6).

### 3.6. MMR Status and Wnt3/β-CTN Indices Across Three-Tier and Binary p53 IHC Classifications

MMR deficiency was more frequent in wild-type p53 tumors (33.3%) than in those with overexpression (2.7%) or null expression (0%), and in the binary classification, it was more frequent in wild-type (33.3%) than in aberrant cases (1.9%) (*p* < 0.001; Supplementary Table S7). The Wnt3 index did not differ among p53 groups, whereas the whole β-CTN index was higher in aberrant p53 tumors (*p* = 0.044), and the nuclear β-CTN index was higher in the p53 overexpression group than in the wild-type (*p* = 0.038; Supplementary Table S7 and Figure 2B).

### 3.7. Associations of Wnt3 and β-CTN Indices with Three-Tier and Binary p53 IHC Classifications

Wnt3 positively correlated weakly to moderately with nuclear β-CTN in the p53 overexpression group (*ρ* = 0.387), with stronger correlations in the p53 null group (*ρ* = 0.507). In the aberrant group, Wnt3 showed weak-to-moderate correlations with nuclear β-CTN (*ρ* = 0.40), whereas in the wild-type group, a weak-to-moderate correlation was observed only with whole β-CTN (*ρ* = 0.374; Supplementary Figure S5). An exploratory analysis of the p53 wild-type group revealed no significant associations between the p53 labeling index and either the Wnt3 or β-CTN expression (Supplementary Figure S6).

### 3.8. Hierarchical Cluster Analysis

**Figure 3:**
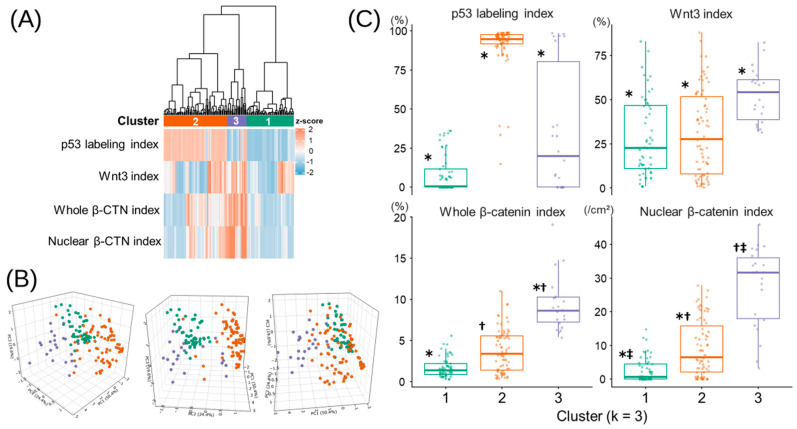
Unsupervised clustering of p53, Wnt3, and β-CTN immunohistochemical profiles. (**A**) Hierarchical clustering heatmap of IHC features. Hierarchical clustering was conducted using z-score-normalized values of the p53 labeling index, whole and nuclear β-CTN indices, and Wnt3 index. Euclidean distance and Ward’s method (Ward.D2) were applied to generate the dendrogram. The heatmap illustrates relative expression levels, with red and blue indicating higher and lower z-scores, respectively. (**B**) Principal component analysis of IHC variables used for hierarchical clustering. The three-dimensional scatter plot shows the spatial distribution of cases according to the three clusters identified (cluster 1, cluster 2, and cluster 3, colored green, orange, and blue, respectively). Each point represents an individual case. The three panels represent the same results viewed from different orientations to facilitate visualization of cluster separation. (**C**) Comparison of IHC features among clusters C1–C3. Boxes indicate the median and interquartile range, with vertical lines denoting the range (minimum–maximum); pairwise *p* values were calculated using Dunn’s test with Bonferroni correction (*, ^†^, *p* < 0.001; ^‡^, *p* = 0.010).

Hierarchical clustering of the overall cohort identified stage-independent immunophenotypic subgroups based on p53 labeling, whole and nuclear β-CTN indices, and Wnt3 (Figure 3A). A three-cluster solution showed better separation than a two-cluster solution, supported by silhouette analysis (mean width: 0.334 for k = 3 vs. 0.274 for k = 2) and the dendrogram, yielding clusters of 53, 71, and 22 cases. Principal component (PC) analysis revealed that PC1, PC2, and PC3 explained 50.4%, 24.4%, and 19.6% of the variance, respectively, and the three clusters were clearly separated in the three-dimensional PC space (Figure 3B). PC1 was mainly driven by whole and nuclear β-CTN indices, PC2 by p53 labeling, and PC3 by Wnt3 expression (Supplementary Table S8). Consistent with these axes, the immunohistochemical indices differed significantly among the three clusters (Figure 3C and Supplementary Table S9).

To assess the biological and clinicopathological relevance of the immunophenotypic clusters, TB and PDC indices and inflammation and desmoplasia scores did not differ among clusters 1–3 (Supplementary Table S10), whereas significant differences were observed in tumor location, the presence of a mucinous component, and MMR status (Supplementary Table S11). In univariate Cox analyses, immunophenotypic clustering was not significantly associated with CSS or OS in the overall cohort, nor with CSS, OS, or RFS in patients with stage I–III disease. In contrast, in stage IV cases, the global test demonstrated a significant difference in survival among clusters (*p* = 0.007), and pairwise comparisons revealed significantly worse survival in cluster 3 compared with clusters 1 and 2 (Supplementary Table S12).

### 3.9. Survival Analysis

For the overall cohort, using cutoffs based on maximally selected rank statistics, univariate Cox analyses identified larger tumor size, higher pT, nodal metastasis, low Wnt3 expression, high TB/PDC indices, and low inflammation scores as adverse factors for CSS. For OS, larger tumor size, nodal positivity, low nuclear β-CTN, and elevated TB and PDC indices (HR 2.40–3.04) were associated with poorer outcomes (Supplementary Table S13). Parsimonious multivariate Cox models, including tumor size, pN category, PDC area index, and inflammation score, were constructed, with baseline covariates selected based on univariable significance (all *p* < 0.05), and each IHC marker was added separately (p53, Wnt3, whole/nuclear β-CTN; Table 2). After adjustment, aberrant p53 showed a trend toward increased mortality (HR: CSS 3.56; OS 2.52), and low Wnt3 tended to be associated with poorer CSS (HR 2.59), whereas neither β-CTN index was prognostic. In all models, the pN and PDC area index remained independently associated with survival.

In 129 patients with stage I–III disease, univariate Cox regression with maximally selected cutoffs identified advanced pT and pN as major adverse predictors of both CSS and RFS. Among histological and immunohistochemical factors, the presence of a mucinous component and high PDC indices, together with low expression of Wnt3 and β-CTN, were associated with adverse outcomes (Supplementary Table S14). A base multivariable Cox model including pT, pN, PDC area index, and inflammation classification was constructed, and representative IHC markers were added individually to assess their independent effects. In the CSS models, advanced pT and pN remained significant predictors, whereas none of the IHC markers reached significance. For RFS, low Wnt3 (HR 2.72), low whole β-CTN (HR 2.52), and low nuclear β-CTN (HR 3.07) emerged as independent adverse factors, while pN consistently retained the strongest prognostic effect across all models (HR 5.90–7.61; Table 3).

In the stage IV subgroup (*n* = 17), fewer lines of salvage chemotherapy were associated with progressively poorer CSS and OS (none vs. ≥2 lines: HR 30.22). A low whole β-catenin index was associated with favorable survival (CSS HR 0.23; OS HR 0.26), whereas a high nuclear β-CTN index predicted significantly worse CSS and OS (both HR ≈ 7.20; Supplementary Table S15).

In the pMMR subgroup (*n* = 131), univariable Cox analysis identified larger tumor size, the presence of a mucinous component, nodal involvement, higher PDC indices, and lower expression of Wnt3 and nuclear β-CTN as significant adverse prognostic factors for survival (Supplementary Table S16). In multivariable Cox models adjusting for tumor size, nodal status, and PDC area index, large tumor size, nodal involvement, and a high PDC area index remained strong and consistent independent predictors of poor outcome. A low nuclear β-CTN index was independently associated with worse OS, whereas low Wnt3 expression showed only a borderline association with poorer CSS (Supplementary Table S17).

### 3.10. Prognostic Value of Composite Scores

**Figure 4:**
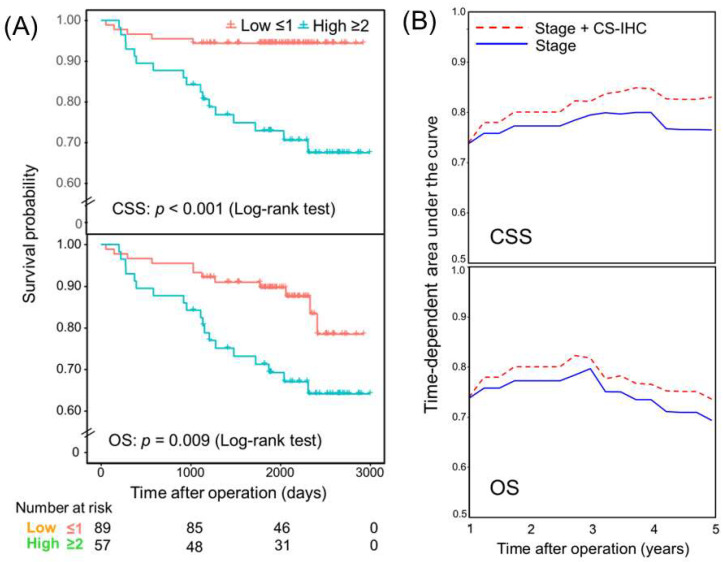
Prognostic impact of pathological stage combined with CS-IHC classification. (**A**) Kaplan–Meier survival curves for CSS and OS according to pathological stage alone and pathological stage combined with the CS-IHC classification. (**B**) Time-dependent ROC analysis showing the time-dependent area under the curve for pathological stage alone and for pathological stage combined with CS-IHC in predicting CSS and OS over time.

Two composite scores (CSs) were constructed: a histomorphological score (CS-HIST) from TB/PDC count indices and an IHC score (CS-IHC) from p53 binary expression and Wnt3/whole β-CTN indices. Each adverse factor was dichotomized (1 = high risk; 0 = low risk), with high risk defined as TB > 6, PDC > 8, aberrant p53, Wnt3 ≤ 27%, and whole β-CTN ≤ 0.6%. The summed score was then assessed for its incremental prognostic value beyond tumor stage. Multivariable Cox regression demonstrated that both CSs were independently associated with CSS and OS after adjustment for stage. Each 1-point increase in CS-IHC was associated with a 2.56-fold and a 1.77-fold higher risk of cancer-specific and overall mortality, respectively. Incorporation of these scores significantly improved model fit compared with the stage-only model, as assessed by the likelihood ratio test (*p* = 0.004 and *p* = 0.036, respectively; Table 4). Kaplan–Meier analyses demonstrated significantly different CSS and OS values according to the pathological stage alone and pathological stage combined with CS-IHC (Figure 4A). Time-dependent ROC analysis showed consistently higher AUC values for the combined model than for stage alone, although the differences were not statistically significant after multiple-testing adjustment (Figure 4B; Supplementary Table S18).

## 4. Discussion

In the present study, both the three-tier and binary p53 IHC classifications were significantly associated with key clinicopathological features. In particular, p53 overexpression was more common in younger patients, left-sided tumors, node-positive cases, and advanced pathological stages than in tumors with a wild-type pattern. Consistent with these findings, tumors classified as p53-positive using a binary IHC threshold (>10–15% nuclear staining) showed a more aggressive phenotype, characterized by poor differentiation, lymphovascular and perineural invasion, deep invasion, nodal involvement, and advanced pathological stage [9,11,14]. Likewise, in stage II and III CRC, aberrant p53 (>10% nuclear staining or a null pattern) has been reported more frequently in left-sided tumors and in stage III cases [10]. We further observed that aberrant p53 expression was associated with non-mucinous histology and smaller tumor size, consistent with previous reports demonstrating a higher prevalence of p53 positivity in non-mucinous than in mucinous CRCs [13]. Notably, CRCs with aberrant p53 tend to present with smaller tumors than those with a wild-type pattern [13], a paradoxical finding that may reflect biological differences in tumor behavior, rather than indicating temporal changes in TP53 alteration during tumor progression.

We found that dMMR occurred in 33% of tumors exhibiting a wild-type p53 pattern but in only 2% of those with aberrant p53 expression, mirroring prior observations that dMMR preferentially associates with wild-type/negative p53 rather than aberrant/positive p53 phenotypes (32% vs. 5%) [13]. Likewise, most MSI-high CRCs displayed a restricted (wild-type-like) p53 staining pattern (84%), whereas most MSS or MSI-low tumors showed aberrant p53 expression (79%) [2]. MSI-high cancers are also frequently associated with mucinous differentiation [15,16]. In our cohort, p53-aberrant tumors were consistent with the chromosome instability-associated spectrum (CMS2-4), where TP53 alterations are common [1]. Wild-type p53 tumors with dMMR corresponded to CMS1 with mucinous features, and the remaining wild-type cases fell within the broader chromosome instability continuum.

A lymphoplasmacytic-predominant infiltrate was present at the invasive front in over 90% of cases, with desmoplasia of variable intensity, consistent with reports that approximately two-thirds of CRCs show a predominantly lymphocytic response at the invasive margin [32]. In this series, inflammatory extent, composition, and desmoplasia did not differ across p53 IHC subgroups, and previous analyses have likewise shown no differences in invasive-front T-cell densities between TP53 wild-type and mutant tumors [38]. Experimental studies suggest that gain-of-function mutant p53 may influence inflammatory signaling and stromal responses [18], although the immune context can vary by molecular subtype; for example, TP53 mutations have been associated with reduced cytotoxic lymphocytes in CMS1 CRCs [3] and in rectal tumors with aberrant p53 expression [37]. In this study, inflammatory patterns were assessed on H&E WSIs without immunophenotypic characterization of immune cell subsets. These routinely available histopathological parameters may nevertheless have potential for clinical risk stratification if validated in independent cohorts.

TB and PDC reflect EMT at the invasive front of CRC [30,31]. Experimental data indicate that disruption of membrane-associated β-CTN complexes promotes its nuclear accumulation and EMT induction, preferentially in TB areas [36], and both features have been linked to nuclear β-CTN [27,28,36]. In the current study, TB and PDC were increased at the invasive front of p53-aberrant tumors, particularly in cases with p53 overexpression and nuclear β-CTN. Loss of wild-type TP53 has been shown to enhance nuclear accumulation of mutant p53 and activate inflammatory and growth factor-related programs, promoting EMT-like and desmoplastic phenotypes [6], thereby providing a biological rationale for the increased TB and PDC in our cohort.

In CRC, Wnt3 is upregulated and may contribute to canonical Wnt signaling involved in tumor progression; its inhibition suppresses pathway activity and delays tumor growth in vivo, yet activity persists despite β-CTN mutations [21,22]. Similarly, Wnt3a is increased in CRC and promotes nuclear β-CTN accumulation and EMT in experimental models [20,25], further stabilizing β-CTN through non-canonical intracellular pathways [24]. Wnt signals cooperate with phosphoinositide-related pathways to drive nuclear β-CTN transcriptional activity [23]. Wild-type p53 upregulates Wnt3, whereas gain-of-function p53 mutations activate β-CTN via phosphorylation-dependent mechanisms [7,19]. Concordant p53/β-CTN expression has also been observed by IHC in CRC [8,12]. In the present study, Wnt3 expression remained comparable across p53 IHC categories, whereas β-CTN activation was significantly increased in aberrant p53 tumors, particularly those with overexpression. Together, these results raise the possibility that p53 dysfunction may be associated with altered canonical Wnt signaling output independently of ligand abundance, thereby favoring nuclear β-CTN accumulation in aberrant tumors.

Additionally, our data revealed a moderate, statistically significant correlation between Wnt3 and nuclear β-CTN in aberrant p53 tumors, contrasting with a weak, non-significant association in the wild-type subset. In wild-type tumors, fluctuations in p53 expression were not linked to measurable changes in Wnt3 or β-CTN levels. This selective strengthening of the Wnt3–β-CTN axis in p53-aberrant tumors suggests that p53 dysfunction, likely reflecting mutant TP53, may be associated with enhanced Wnt pathway activation; wild-type p53 imposes regulatory restraint. The gain-of-function mutant p53 enhances β-CTN-dependent transcription through phosphorylation-dependent mechanisms [19]. By contrast, wild-type p53 restrains Wnt signaling by promoting β-CTN turnover and secreted antagonists [7]. Gain-of-function TP53 missense mutations have also been proposed to activate transcription factors, including Wnt/β-catenin, through altered transcriptional regulation, particularly after wild-type p53 loss [6].

Hierarchical clustering of p53, β-CTN, and Wnt3 indices separated tumors into three subsets aligned with distinct PCs: C1 showed lower β-CTN levels, C2 was enriched for p53 labeling, and C3 displayed higher Wnt3 expression. Although Wnt3 levels varied little across p53 categories, nuclear β-CTN accumulation was selectively enhanced in p53-aberrant tumors, particularly those with overexpression, and a significant Wnt3–β-CTN correlation emerged only in this subset, not in wild-type cases. Evidence supports a bidirectional regulatory relationship between TP53 and the Wnt pathway, whereby mutant TP53 can potentiate β-CTN-driven transcription and wild-type p53 constrains pathway output [6,7,19]. In CRC, Wnt3 acts as an upstream determinant of Wnt signaling, reinforcing pathway activation and associating with nuclear β-CTN accumulation [20,21,22,25]. Thus, while β-CTN, p53, and Wnt3 segregate across principal components, their context-dependent correlations and documented interplay suggest coordinated functional interaction.

These PC-defined clusters were not significantly associated with TB, PDC, inflammation, or desmoplasia. Although p53-aberrant tumors, particularly those with overexpression, exhibited increased TB and PDC individually, this association was not retained at the cluster level, and inflammatory and desmoplastic features did not vary consistently across p53 categories. Mechanistically, gain-of-function mutant TP53 promotes pro-inflammatory transcription and stromal remodeling, amplified by loss of the remaining wild-type TP53 allele [6,18], suggesting that invasive-front and stromal features reflect spatially restricted microenvironmental influences rather than global tumor-intrinsic signaling states. The C1 cluster, aligned with the β-CTN axis, was enriched for dMMR and mucinous differentiation. Increased MSI-high frequency together with reduced nuclear β-CTN labeling has been described in mucinous CRC [15], and the association with dMMR aligns with prior observations linking reduced β-CTN expression to MLH1 loss [8]. Together, this distribution indicates that C1 overlaps recognized MSI-associated and mucinous phenotypes.

Survival analyses showed heterogeneous prognostic patterns across stages and molecular subsets. In the overall cohort, nodal status and PDC burden were the principal survival determinants, whereas IHC-based signaling markers added limited value after adjustment, with aberrant p53 showing borderline association with poorer outcome. However, aberrant p53 expression was significantly associated with aggressive tumor features, including nodal metastasis and increased TB/PDC indices, suggesting that p53 aberration reflects tumor aggressiveness even if it does not function as an independent prognostic factor in multivariable models. Prior studies report heterogeneous prognostic effects of p53 IHC in CRC, some identifying overexpression or complete loss as adverse features [5,9,11,13], while others did not confirm independent significance [8,10]. In stage I–III disease, this hierarchy persisted for CSS, although low Wnt3 and reduced β-CTN were linked to recurrence. In stage IV tumors, prognosis was largely driven by treatment, and lower whole β-CTN was associated with adverse outcome in this subgroup. Among pMMR cancers, decreased nuclear β-CTN retained independent prognostic relevance, yet established pathological factors remained the dominant survival influences.

Evidence linking Wnt3 or Wnt3a and β-CTN IHC to clinical outcomes in CRC remains limited and inconsistent. Increased Wnt3a expression has been associated with poorer survival [20], although this has not been consistently validated [25,26] and contrasts with our finding that reduced Wnt3 levels were related to recurrence. In our cohort, Wnt3 staining was predominantly stronger in tumor cells than in adjacent non-neoplastic epithelium and gradually decreased toward deeper invasive regions. This spatial gradient suggests that Wnt3 expression may reflect tumor–microenvironment interactions or changes in tumor differentiation during invasion rather than direct ligand-dependent activation of canonical Wnt signaling. Therefore, the association between lower Wnt3 expression and adverse prognosis should be interpreted cautiously.

For β-CTN, prognostic impact varies by subcellular localization, and reduced membranous expression at the invasive front has been linked to poor survival [27], though not uniformly across cohorts [12,26,28]. In contrast, nuclear β-CTN overexpression has been identified as an independent adverse factor [25], with meta-analytic evidence supporting worse survival across endpoints [29]. We found that the relatively low median value of the whole β-CTN index reflects that its expression in tumor cells was often comparable to or weaker than that in adjacent non-neoplastic epithelium, particularly at the invasive front where TB and PDC are enriched. Further, the low nuclear β-CTN index independently predicted recurrence in stage I–III disease and was associated with worse overall survival in proficient MMR tumors. One technical consideration is that nuclear β-CTN counts were normalized to the whole β-CTN-positive tumor area derived from threshold-based image analysis rather than to the total epithelial tumor area. Because membranous β-CTN staining was often reduced at the invasive front, tumor areas with attenuated β-CTN expression may have been underestimated. Another possible explanation is that invasive-front tumor cells undergoing EMT may exhibit reduced membranous expression and heterogeneous nuclear signaling, resulting in an overall lower nuclear β-CTN index despite biologically aggressive behavior.

The CSs were designed for clinical applicability, using simplified count-based thresholds for TB and PDC, binary p53 classification, and image-based quantification of Wnt3 and whole β-CTN to enhance reproducibility in routine practice. Although individual IHC markers were not independently prognostic in multivariable analysis, CS-IHC, together with CS-HIST, provided independent prognostic value beyond the pathological stage, with incremental increases in CS-IHC associated with stepwise rises in CSS and OS and improved model fit compared with stage alone. Integration of tumor signaling markers within the p53–Wnt/β-CTN axis may therefore provide incremental prognostic information beyond standard pathological staging.

Several limitations warrant consideration. This retrospective single-institution study included a moderate sample size, and subgroup analyses, particularly in stage IV disease, were limited by small numbers. Cutoff values for continuous variables were derived using maximally selected rank statistics within the cohort, which may introduce data-driven bias and increase the risk of Type I errors in studies with modest sample sizes. Although the CSs improved model fit beyond the pathological stage, their incremental discriminatory gain was modest and requires validation in independent cohorts. Finally, the analysis relied on immunohistochemical surrogates of signaling activity without direct genomic or transcriptomic integration, which may limit biological interpretation. MMR status was assessed using a two-antibody panel (PMS2 and MSH6), which does not fully replicate the conventional four-marker panel or MSI-based testing. BRAF mutation analysis was not performed in most dMMR cases. Because the number of dMMR tumors in this cohort was limited, a small degree of misclassification cannot be excluded, and MMR-related subgroup findings should be interpreted with caution. In addition, TP53 mutation status was not directly validated by sequencing. Although aberrant p53 immunostaining patterns are widely used as surrogate markers of TP53 alterations, IHC cannot precisely determine the exact mutation type or functional consequences, and therefore, the findings reflect p53 immunophenotypes rather than mutation status. Also, interpretations regarding TP53-driven signaling interactions in this study should be considered hypothesis-generating and exploratory. Future studies integrating genomic datasets such as The Cancer Genome Atlas and multichannel immunofluorescence may help further clarify the relationship between TP53 alterations and Wnt/β-CTN signaling.

## 5. Conclusions

CRCs with aberrant p53 expression exhibited more advanced pathological features and increased EMT-associated TB and PDC compared with wild-type tumors and were largely confined to the pMMR subgroup. Nuclear β-CTN accumulation was preferentially observed in aberrant p53 tumors, independent of overall Wnt3 levels, indicating functional dissociation within the p53–Wnt/β-CTN axis, a pattern supported by integrated analysis. Although conventional pathological parameters remained the dominant survival determinants, the prognostic impact of Wnt/β-CTN signaling varied across clinical subgroups, and an immunohistochemistry-based composite score integrating these markers added prognostic value beyond stage, underscoring the potential of combining signaling markers with conventional staging to improve risk stratification.

## Figures and Tables

**Table 1:** Comparison of clinicopathological variables according to binary and three-tier p53 immunohistochemical classifications.

Variable	p53 Three-Tier			*p* Value	p53 Binary	*p* Value
	Overexpression	Null	Wild	(3 Groups)	Aberrant	(vs. Wild)
*n* (%)	73 (50.0)	34 (23.3)	39 (26.7)		107 (73.3)	
Age [years]						
≤70	33 (45.2) ^†^	8 (23.5)	7 (17.9) ^†^	0.006 *	41 (38.3)	0.028 *
>70	40 (54.8)	26 (76.5)	32 (82.1)		66 (61.7)	
Sex						
Male	49 (67.1)	21 (61.8)	18 (46.2)		70 (65.4)	0.055
Female	24 (32.9)	13 (38.2)	21 (53.8)	0.095	37 (34.6)	
Location						
Left	50 (68.5) ^†^	24 (70.6) ^‡^	21 (53.8) ^†, ‡^	<0.001 *	74 (69.2)	<0.001 *
Right	23 (31.5)	10 (29.4)	18 (46.2)		33 (30.8)	
Size [mm]						
>40	38 (52.1)	17 (50.0)	25 (64.1)	0.085	55 (51.4)	0.038 *
≤40	35 (47.9)	17 (50.0)	14 (35.9)		52 (48.6)	
Differentiation						
Well	34 (46.6)	11 (32.4)	16 (41.0)	0.378	45 (42.1)	1.000
Mod. + Poor	39 (53.4)	23 (67.6)	23 (59.0)		62 (57.9)	
Mucinous						
Absent	59 (80.8)	30 (88.2)	26 (66.7)	0.066	89 (83.2)	0.040 *
Present	14 (19.2)	4 (11.8)	13 (33.3)		18 (16.8)	
pT						
1 + 2	24 (32.9)	7 (20.6)	12 (30.8)	0.421	31 (29.0)	0.840
3 + 4	49 (67.1)	27 (79.4)	27 (69.2)		76 (71.0)	
pN						
0	39 (53.4) ^†^	18 (52.9)^‡^	24 (61.5) ^†, ‡^	0.007 *	57 (53.3)	0.002 *
1 + 2	34 (46.6)	16 (47.1)	15 (38.5)		50 (46.7)	
Stage						
I + II	38 (52.1) ^†^	18 (52.9) ^‡^	24 (61.5) ^†, ‡^	0.013 *	56 (52.3)	0.004 *
III + IV	35 (47.9)	16 (47.1)	15 (38.5)		51 (47.7)	

p53 three-tier: Overexpression, null, and wild-type; p53 binary classification consisted of aberrant (overexpression or null) and wild type. Differentiation: well, well-differentiated tubular adenocarcinoma; mod., moderately differentiated; poor, poorly differentiated; mucinous, presence of mucinous component within the tumor. ^†^ Significant differences between overexpression and wild-type for age (Holm-adjusted *p* = 0.015), tumor location (*p* = 0.001), pN category (*p* = 0.019), and stage (*p* = 0.004). ^‡^ Significant differences between null-type and wild-type for location (*p* = 0.015), pN (*p* = 0.021), and stage (*p* = 0.018). Patients with aberrant p53 were younger (median 75 [interquartile range: 64–83] vs. 80 [72–84] years; *p* = 0.017) and had smaller tumors (median 38 [25.5–55] vs. 45 [29.5–76] mm; *p* = 0.027, Mann–Whitney *U* test). Data are expressed as a number (percentage); * *p* < 0.05.

**Table 2:** Multivariate Cox proportional hazards analyses for CSS and OS in the overall cohort.

Variable (Comparison Level/vs. Reference)	CSS			OS	
	HR (95%CI)	*p* Value		HR (95%CI)	*p* Value
Model 1					
Tumor size [mm] (≥40 vs. <40)	5.088 (1.774–14.598)	0.002 *		3.090 (1.366–6.988)	0.007 *
pN (pN1 + 2 vs. pN0)	9.525 (3.109–29.184)	<0.001 *		3.390 (1.586–7.247)	0.002 *
PDC area index [/μm^2^] (high >9000 vs. low ≤9000)	4.525 (1.748–11.628)	0.002 *		3.690 (1.650–8.264)	0.001 *
Inflammation score (low ≤5 vs. high >5)	3.363 (0.764–14.809)	0.109		1.985 (0.742–5.312)	0.172
p53 binary classification (aberrant vs. wild)	3.560 (0.811 –15.625)	0.092		2.519 (0.857–7.407)	0.093
Model 2					
Tumor size [mm] (≥40 vs. <40)	3.593 (1.219–10.591)	0.020 *		2.209 (0.958–5.095)	0.063
pN (pN1 + 2 vs. pN0)	10.202 (3.338–31.181)	<0.001 *		3.611 (1.697–7.683)	<0.001 *
PDC area index [/μm^2^] (high >9000 vs. low ≤9000)	4.837 (1.860–12.576)	0.001 *		3.924 (1.758–8.756)	<0.001 *
Inflammation score (low ≤5 vs. high >5)	2.830 (0.626–12.788)	0.176		1.863 (0.688–5.048)	0.221
Wnt3 index [%] (low ≤27 vs. high >27)	2.588 (0.970–6.907)	0.058		1.488 (0.699–3.170)	0.303
Model 3					
Tumor size [mm] (≥40 vs. <40)	4.131 (1.455–11.726)	0.008 *		2.598 (1.165–5.793)	0.020 *
pN (pN1 + 2 vs. pN0)	9.996 (3.268–30.575)	<0.001 *		3.612 (1.692–7.709)	<0.001 *
PDC area index [/μm^2^] (high >9000 vs. low ≤9000)	4.666 (1.764–12.341)	0.002 *		3.903 (1.734–8.789)	0.001 *
Inflammation score (low ≤5 vs. high >5)	3.063 (0.685–13.692)	0.143		1.993 (0.742–5.356)	0.171
Whole β-CTN index [%] (low ≤0.6 vs. high >0.6)	1.165 (0.377–3.596)	0.791		0.996 (0.339–2.927)	0.994
Model 4					
Tumor size [mm] (≥40 vs. <40)	3.190 (1.090–9.337)	0.034 *		2.397 (1.066–5.391)	0.035 *
pN (pN1 + 2 vs. pN0)	10.048 (3.297–30.624)	<0.001 *		3.609 (1.700–7.659)	<0.001 *
PDC area index [/μm^2^] (high >9000 vs. low ≤9000)	4.310 (1.656–11.236)	0.003 *		3.636 (1.610–8.197)	0.002 *
Inflammation score (low ≤5 vs. high >5)	4.059 (0.883–18.649)	0.072		2.159 (0.795–5.860)	0.131
Nuclear β-CTN index (≤0.5 vs. >0.5)	1.696 (0.688–4.180)	0.251		1.792 (0.829–3.871)	0.138

CSS, Cancer-specific survival; OS, overall survival; HR, hazard ratio; CI, confidence interval; p53 binary classification consisted of aberrant (overexpression or null) and wild type; PDC, poorly differentiated cluster; β-CTN, β-catenin; * *p* < 0.05.

**Table 3:** Multivariate Cox proportional hazards analyses for RFS in the stage I-III subgroup.

Variable (Comparison Level/vs. Reference)	CSS			RFS	
	HR (95%CI)	*p* Value		HR (95%CI)	*p* Value
Model 1					
pT (pT3 + 4 vs. pT1 + 2)	10.773 (1.367–1389.510)	0.018 *		3.744 (1.095–12.804)	0.035 *
pN (pN1 + 2 vs. pN0)	3.812 (1.100–16.367)	0.034 *		7.144 (2.584–19.747)	<0.001 *
PDC area index [/μm^2^] (high >3000 vs. low ≤3000)	3.111 (0.672–29.877)	0.160		2.968 (0.908–9.698)	0.072
Inflammation classification (3 groups)	0.929 (0.349–2.371)	0.097		1.276 (0.483–3.371)	0.623
p53 binary classification (aberrant vs. wild)	1.275 (0.250–12.615)	0.790		0.759 (0.221–2.604)	0.661
Model 2					
pT (pT3 + 4 vs. pT1 + 2)	11.549 (1.447–1494.192)	0.015 *		3.744 (1.095–12.804)	0.035 *
pN (pN1 + 2 vs. pN0)	4.624 (1.378–19.182)	0.013 *		7.612 (2.824–20.523)	<0.001 *
PDC area index [/μm^2^] (high >3000 vs. low ≤3000)	0.271 (0.029–1.172)	0.085		2.841 (0.934–8.621)	0.072
Inflammation classification (3 group)	1.008 (0.375–2.618)	0.100		1.452 (0.530–3.976)	0.468
Wnt3 index [%] (low ≤5.0 vs. high >5.0)	0.369 (0.111–1.527)	0.154		2.720 (1.037–7.136)	0.042 *
Model 3					
pT (pT3 + 4 vs. pT1 + 2)	9.950 (1.260–1284.217)	0.024 *		3.466 (1.010–11.888)	0.048 *
pN (pN1 + 2 vs. pN0)	4.188 (1.249–17.340)	0.020 *		6.403 (2.428–16.886)	<0.001 *
PDC area index [/μm^2^] (high >3000 vs. low ≤3000)	0.308 (0.033–1.360)	0.131		2.711 (0.908–8.130)	0.074
Inflammation classification (3 groups)	0.897 (0.339–2.284)	0.094		0.928 (0.341–2.526)	0.883
Whole β-CTN index [%] (≤1.8 vs. >1.8)	0.623 (0.186–2.026)	0.426		2.518 (1.076–5.892)	0.033 *
Model 4					
pT (pT3 + 4 vs. pT1 + 2)	9.443 (1.184–1221.969)	0.030 *		3.189 (0.922–11.030)	0.067
pN (pN1 + 2 vs. pN0)	3.948 (1.170–16.430)	0.026 *		5.898 (2.229–15.608)	<0.001 *
PDC area index [/μm^2^] (high >3000 vs. low ≤3000)	0.261 (0.028–1.152)	0.080		3.584 (1.178–10.869)	0.025 *
Inflammation classification (3 groups)	0.874 (0.318–2.293)	0.095		1.012 (0.377–2.713)	0.981
Nuclear β-CTN index [/cm^2^] (≤6.6 vs. >6.6)	0.613 (0.146–2.098)	0.447		3.073 (1.118–8.449)	0.030 *

RFS, Recurrence-free survival; HR, hazard ratio; CI, confidence interval; PDC, poorly differentiated cluster; β-CTN, β-catenin. Inflammation was subclassified according to the predominant inflammatory cell type as lymphoplasmacytic and granulocytic (neutrophilic and eosinophilic) or histiocytic; p53 binary classification consisted of aberrant (overexpression or null) and wild type; * *p* < 0.05.

**Table 4:** Multivariable Cox regression analysis of composite scores (CS-HIST and CS-IHC) for CSS and OS.

Model	CSS				OS		
	HR (95% CI)	*p* Value	LR *p* Value (vs. Stage)		HR (95% CI)	*p* Value	LR *p* Value (vs. Stage)
Stage + CS-HIST	2.308 (1.153–4.622)	0.018 *	0.018 *		2.028 (1.165–3.530)	0.012 *	0.013 *
Stage + CS-IHC	2.561 (1.318–4.976)	0.006 *	0.004 *		1.773 (1.037–3.031)	0.036 *	0.033 *
Stage + CS-HIST + IHC			0.004 *				0.016 *
Stage + CS-HIST	1.842 (0.884–3.837)	0.103			1.785 (1.001–3.184)	0.05	
Stage + CS-HIST/IHC	2.254 (1.121–4.530)	0.023 *			1.537 (0.870–2.715)	0.139	

CS, Composite score; CS-HIST, composite score constructed from TB (tumor budding) and PDC (poorly differentiated cluster) indices; CS-IHC, composite score constructed from p53 binary expression, Wnt3 index, and whole β-CTN (β-catenin); CSS, cancer-specific survival; OS, overall survival; HR, hazard ratio; CI, confidence interval; LR, likelihood ratio; HRs (95% CIs) indicate the risk associated with a 1-point increase in the composite score (CS); * *p* < 0.05.

## Data Availability

The data that support the findings of this study are not publicly available because they contain information that could compromise the privacy of research participants, but they are available from the corresponding author, S.C., upon reasonable request.

## Supplementary Materials

The following supporting information can be downloaded at https://www.mdpi.com/article/10.3390/curroncol33030178/s1. Figure S1: Study flow diagram of patient selection and exclusion criteria; Figure S2: Representative histological assessment of inflammation and desmoplasia at the invasive front; Figure S3: Correlation between whole and nuclear β-CTN indices; Figure S4: Pairwise comparisons of clinicopathological variables according to the three-tier p53 IHC classification; Figure S5: Spearman’s correlations between Wnt3 and β-CTN indices according to p53 IHC classification; Figure S6. Correlations between the p53 labeling index and Wnt3 and β-CTN indices in the p53 wild-type group. Table S1: Patient characteristics and follow-up outcomes; Table S2: Summary of chemotherapy regimens in the adjuvant and salvage settings; Table S3: Clinicopathological features of the overall cohort; Table S4: Clinicopathological features of the subgroups; Table S5: Comparison of TB and PDC indices according to three-tier and binary p53 immunohistochemical classifications; Table S6: Comparison of inflammation and desmoplasia scores according to three-tier and binary p53 immunohistochemical classifications; Table S7: Comparison of IHC assessment according to three-tier and binary p53 immunohistochemical classifications; Table S8: Principal component loadings of immunohistochemical variables used for hierarchical clustering; Table S9: Comparison of immunohistochemical features among three hierarchical clusters based on p53, Wnt3, and β-CTN expression; Table S10: TB, PDC, and inflammation and desmoplasia scores according to immunophenotypic clusters; Table S11: Clinicopathological characteristics and MMR system status according to immunophenotypic clusters; Table S12: Univariate Cox regression for survival in the overall cohort and stage-based subgroups according to immunophenotypic clustering; Table S13: Univariate Cox regression analysis for CSS and OS in the overall cohort; Table S14: Univariate Cox regression analysis for RFS in the stage I-III subgroup; Table S15: Univariate Cox regression analysis for CSS in the stage IV subgroup; Table S16: Univariate Cox regression analysis for CSS and OS in the pMMR subgroup; Table S17: Multivariate Cox proportional hazards analyses for CSS and OS in the pMMR group; Table S18: Time-dependent AUC comparison between pathological stage alone and pathological stage combined with CS-IHC for CSS and OS; File S1: Supplementary Methods.

## Abbreviations

The following abbreviations are used in this manuscript:

AUC	Area Under the Curve;
CI	Confidence Interval;
CMS	Consensus Molecular Subtype;
CRC	Colorectal Cancer;
CS	Composite Score;
CSS	Cancer-Specific Survival;
EMT	Epithelial–Mesenchymal Transition;
H&E	Hematoxylin and Eosin;
HR	Hazard Ratio;
IHC	Immunohistochemistry;
MMR	Mismatch Repair;
MSI	Microsatellite Instability;
MSS	Microsatellite Stable;
OS	Overall Survival;
PDC	Poorly Differentiated Cluster;
RFS	Recurrence-Free Survival;
ROC	Receiver Operating Characteristic;
TB	Tumor Budding;
UGT1A1	Uridine Diphosphate Glucuronosyltransferase 1A1;
WSI	Whole-Slide Imaging.
