# Supplementary material for: Interplay Between p53 and Wnt/β-Catenin Signaling in Colorectal Cancer: Associations with Mismatch Repair Status, Tumor Microenvironment, and Clinicopathological Outcomes
Source: Curr Oncol. 2026 Mar 21;33(3):178. doi: 10.3390/curroncol33030178 (PMC13025743; doi:10.3390/curroncol33030178)
Supplement: Supplementary file 1 [file curroncol-33-00178-s001.zip › Supplementary File S1.zip!/Supplementary Methods.pdf]

## Supplementary Methods

All image analyses were conducted using ImageJ/Fiji (National Institutes of Health, Bethesda, MD, USA; https://fiji.sc/) and digital whole-slide images (WSIs) were reviewed with NDP.view2 (v2.9.29; Hamamatsu Photonics, Hamamatsu, Japan).

### 1. Image-based evaluation of tumor budding and poorly differentiated clusters

*Definition of tumor budding and poorly differentiated clusters*

Tumor budding (TB) was defined as a single tumor cell or a cluster consisting of one to four tumor cells. Poorly differentiated cluster (PDC) was defined as clusters composed of five to ten tumor cells, distinct from conventional TB. TB and PDC were evaluated on H&E–stained sections and were considered distinct histopathological entities.

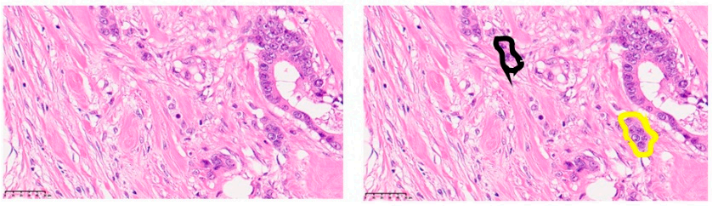

Figure SM1. Manual annotation of TB and PDC.

Representative H&E-stained image of the invasive front before annotation (left) and the same field after manual annotation (right), showing a single TB (black outline) and a single PDC (yellow outline).

*Software*

ImageJ/Fiji was used for quantification of tumor budding (TB) and poorly differentiated clusters (PDC).

*Image acquisition*

WSIs of H&E-stained sections were reviewed to identify representative areas, which were subsequently captured at ×40 magnification and exported as JPEG files.

*Image analysis procedure*

Each JPEG image was opened in ImageJ/Fiji (File → Open). The ROI Manager was activated prior to annotation to enable systematic registration of regions of interest (Analyze → Tools → ROI Manager).

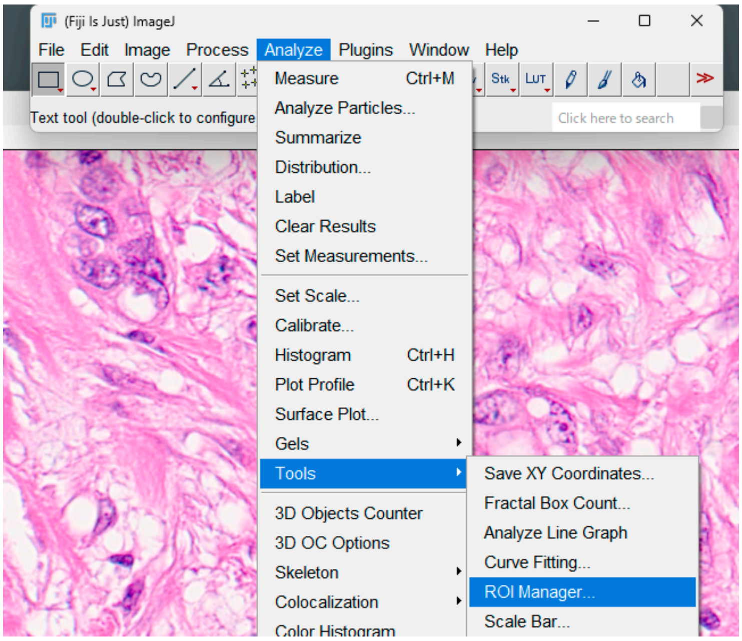


Figure SM2. Activation of the ROI Manager.

Screenshot of the ImageJ/Fiji interface showing activation of the ROI Manager to enable systematic registration and management of regions of interest prior to annotation.

Spatial calibration was performed by tracing a straight line along the reference scale bar using the *Straight Line* tool, followed by Analyze > Set Scale. The known distance (50) and unit of length (μm) were entered, and the scale was applied uniformly across all images.

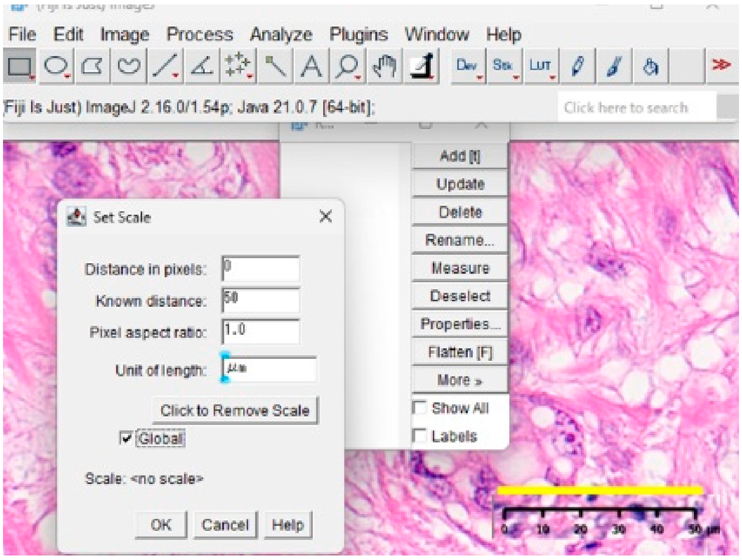


Figure SM3. Spatial calibration using the reference scale bar.

Spatial calibration was performed by drawing a straight line (yellow line) along the reference scale bar and entering the known distance to standardize measurements across images.

Using the *Freehand Selection* tool, individual TB (labeled #1) and PDC (labeled #2) were manually traced separately. All annotated regions were added to the ROI

Manager, allowing visualization of all selected regions and their corresponding labels (#1 and #2), as displayed in the ROI Manager.

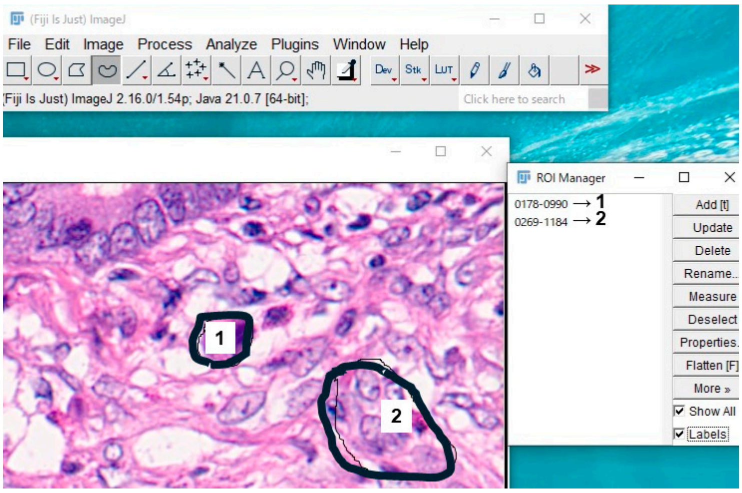


Figure SM4. Manual tracing of individual TB and PDC.

Individual TB and PDC were manually traced using the Freehand Selection tool, and each annotated region was sequentially added to the ROI Manager.

For each annotated region, measurements were obtained by selecting the corresponding ROIs and applying the Measure function (yellow arrow). The Results window displayed the measured area for each annotated TB or PDC (red box), reported in square micrometers (μm²; blue box).

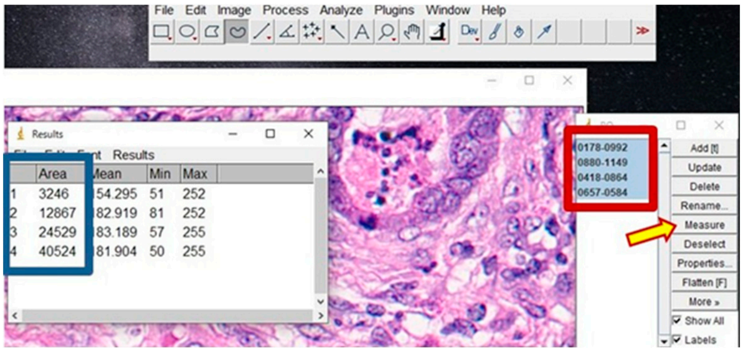

Figure SM5. Measurement of annotated regions.

Measurement of manually annotated TB and PDC using the Measure function in ImageJ/Fiji, showing the output of area values for each individual region in the Results window.

Measurement results were exported as comma-separated value (CSV) files (File → Save As → CSV). For each case, five representative high-power fields at ×40 objective were assessed, and four quantitative indices were defined: the TB count index (total number of TBs in five high-power fields), the TB area index (total area [μm²] occupied by TBs per five high-power fields), the PDC count index (total number of PDCs in five high-power fields), and the PDC area index (total area [μm²] occupied by PDCs per five high-power fields).

*Quality control*

All annotations were performed by one investigator (S.C.) and independently reviewed by another investigator (H.M.). Any discrepancies in the identification or classification of TBs and PDCs were resolved by joint review and consensus. All images were analyzed using identical parameter settings to ensure consistency and reproducibility.

**2. p53 nuclear count**

*Software*

ImageJ/Fiji was used for quantification of p53-positive tumor nuclei using the Cell Counter plugin.2.

*Image acquisition*

ImageJ/Fiji was used for quantification of p53-positive tumor nuclei using the Cell Counter plugin.

*Image analysis procedure*

Counting procedure: Open each JPEG file in ImageJ/Fiji.

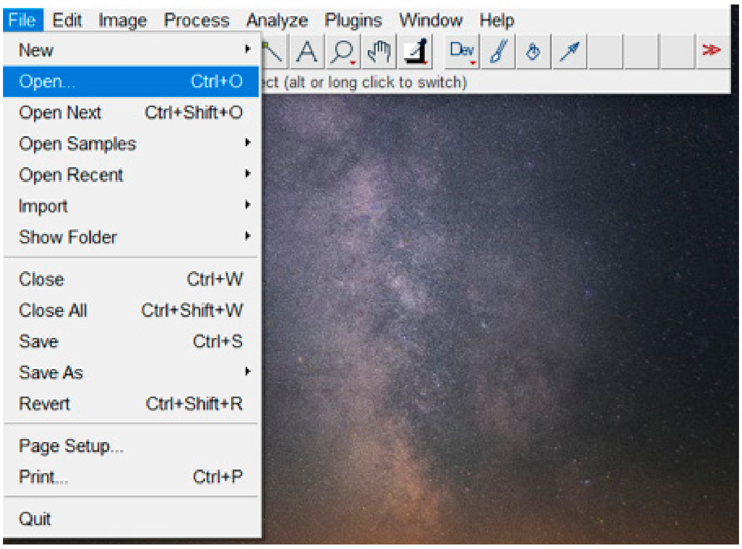


Figure SM6. Opening JPEG images in ImageJ/Fiji.

Screenshot of the ImageJ/Fiji interface showing the “File → Open” command used to load JPEG images for analysis.

Launch the Cell Counter plugin.

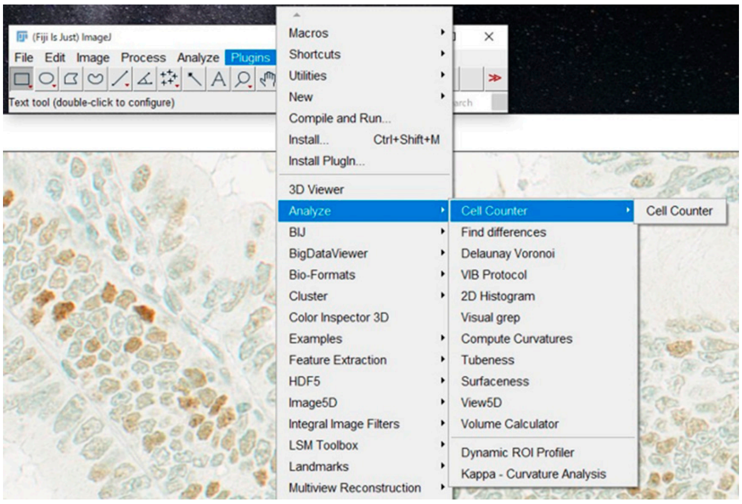


Figure SM7. Launching the Cell Counter plugin.

Screenshot of the ImageJ/Fiji interface showing access to the Cell Counter plugin via the “Plugins → Analyze → Cell Counter” menu.

Select Keep original → Initialize → Show Numbers to begin manual annotation.

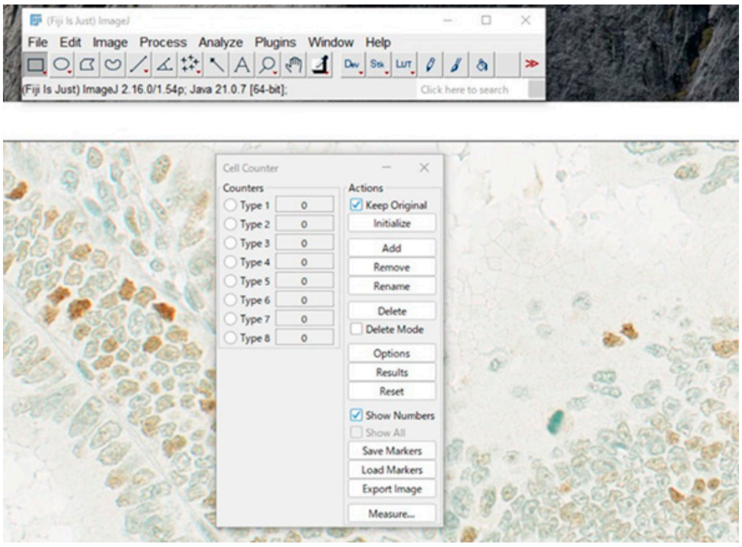


Figure SM8. Cell Counter panel and manual nuclear marking.

Screenshot of the Cell Counter panel showing available marker types and action buttons. Each positively stained tumor nucleus was manually marked using the designated marker tool.

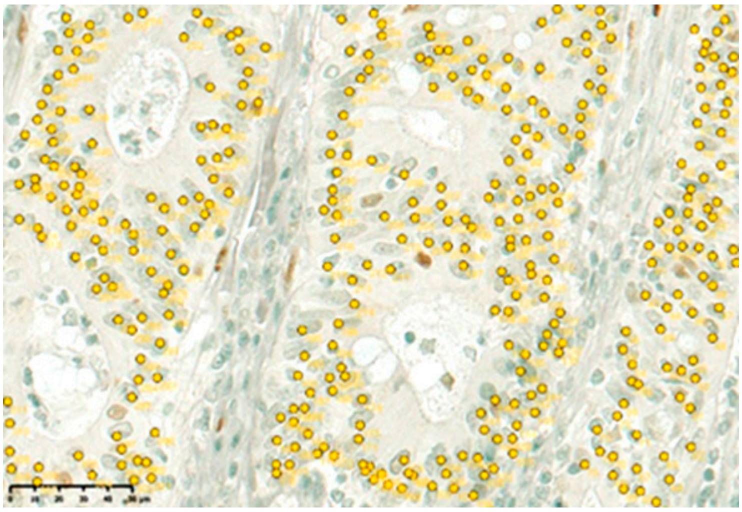

Figure SM9. Manual marking of p53-positive tumor nuclei. Representative image showing p53-positive tumor nuclei manually marked with yellow circles using the Cell Counter plugin.

The total count was displayed in the *Results* window (indicated by the red box on the left), which could be opened by clicking the *Results* button (yellow arrow) in the *Cell Counter* panel.

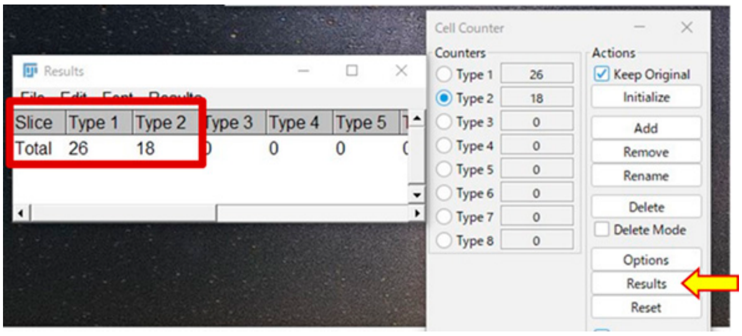


Figure SM10. Display of total nuclear count in the Results window.

Screenshot showing the total count displayed in the Results window (red box, left), which can be opened by clicking the Results button (yellow arrow) in the Cell Counter panel.

*Calculation*

For each case, more than 1000 tumor nuclei were counted to calculate the p53 labeling index (%) by one investigator (H.M.) using this plugin function.

$$\text{p53 labeling index (\%)} = \frac{\text{number of immunoreactive nuclei}}{\text{total counted nuclei}} \times 100$$

*Quality control*

Another investigator (S.C.) independently evaluated the staining pattern by visual inspection. Any discrepancies in nuclear count or staining interpretation were resolved through joint review and consensus.

**3. Wnt3 index quantification**

*Software*

ImageJ/Fiji was used for quantitative analysis of Wnt3 immunohistochemistry.

*Image acquisition*

WSIs of Wnt3-stained sections were acquired at ×0.63 magnification to include both tumor and adjacent non-neoplastic mucosa. Representative JPEG images were exported for quantitative analysis.

***Preliminary study for threshold determination***

Prior to the main analysis, a preliminary study was conducted to determine appropriate threshold values for Wnt3 signal detection, a preliminary analysis was conducted using 10 randomly selected cases.

*Image acquisition*

Each JPEG image was opened in ImageJ/Fiji.

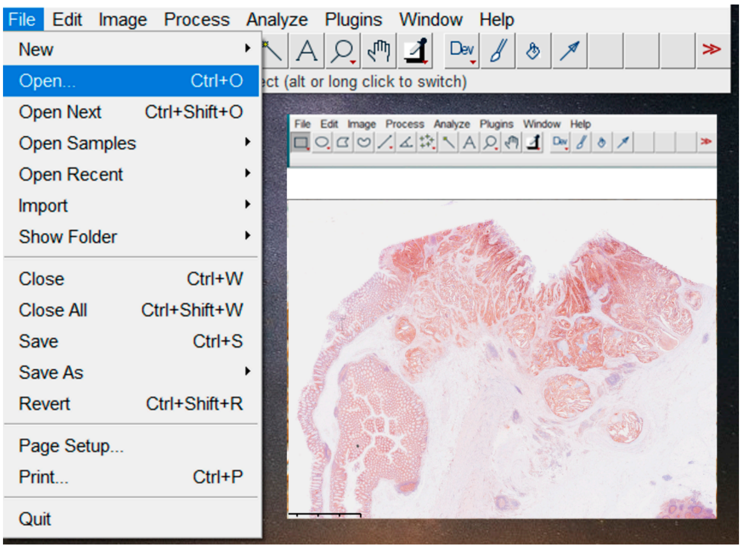


Figure SM11. Opening a JPEG image in ImageJ/Fiji.

Screenshot of the ImageJ/Fiji interface showing a JPEG image opened for quantitative analysis.

*Image preprocessing*

Images were converted to 8-bit grayscale format to standardize signal intensity values across cases.

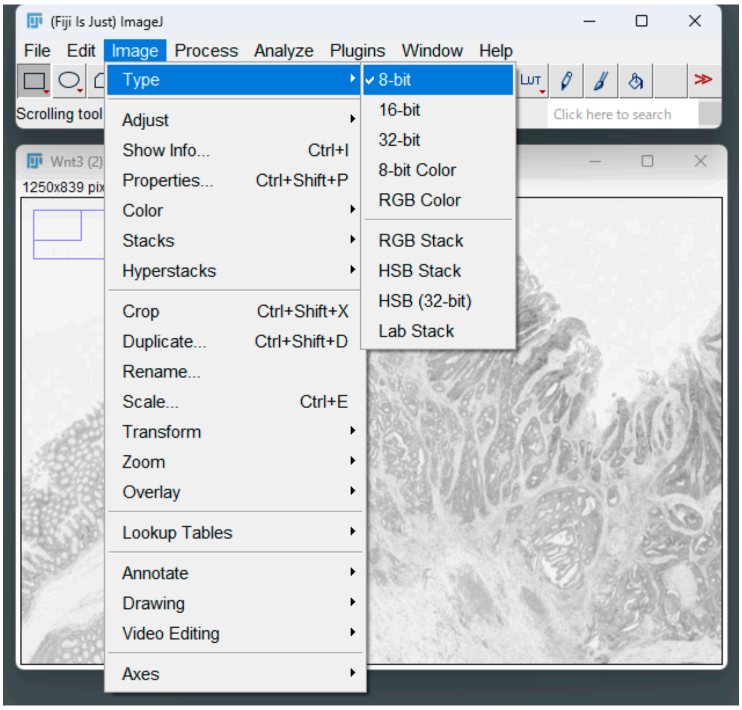


Figure SM12. Conversion to 8-bit grayscale.

Images were converted to 8-bit grayscale format in ImageJ/Fiji to standardize signal intensity values across cases prior to threshold-based analysis.

***Initial thresholding***

Thresholding was applied to segment Wnt3-positive signals from background tissue. The lower threshold was fixed at 0 in all images to eliminate background noise and ensure consistent baseline signal suppression. Using this standardized preprocessing and thresholding approach, Wnt3 immunoreactivity in tumor tissue was systematically compared with that in adjacent non-neoplastic mucosa across a randomly selected subset of 10 cases.

(i) The tumor-specific upper threshold was defined as the minimum intensity that largely excluded non-neoplastic signals while preserving tumor-specific staining. This value ranged from 165 to 185 across 10 cases (mean, 175).

(ii) The entire-tumor upper threshold was defined to encompass the whole tumor epithelium while minimizing stromal staining. This value ranged from 190 to 215, with a mean of 200, and was adopted as the higher upper threshold. Based on this preliminary evaluation, these threshold values were applied to subsequent analyses.

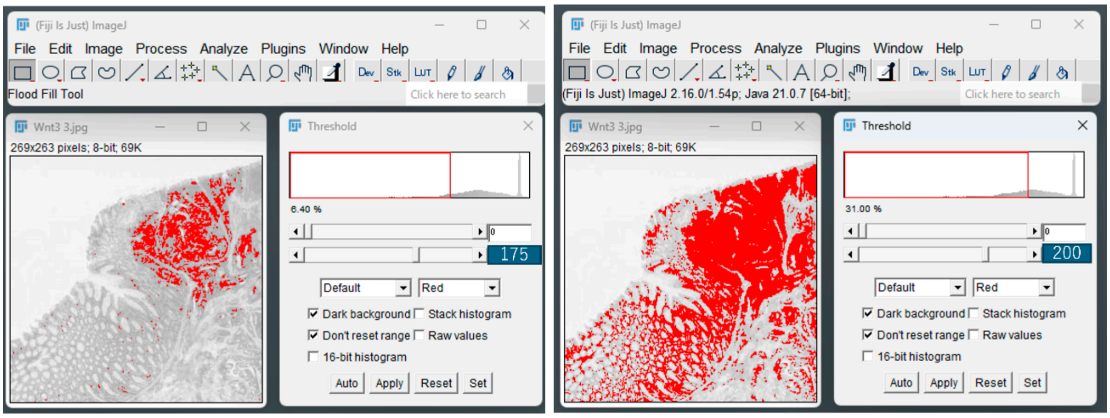


Figure SM13. Preliminary threshold determination for Wnt3 immunoreactivity.

Representative images showing stepwise adjustment of lower and upper threshold values based on comparison between tumor tissue and adjacent non-neoplastic mucosa to define optimal segmentation parameters.

### ***All-case image analysis***

*Tumor region annotation*

Tumor regions were annotated with reference to serial H&E-stained WSIs. The corresponding tumor area on the Wnt3-stained WSI was manually outlined using the *Freehand Selection* tool and saved in the *ROI Manager*.

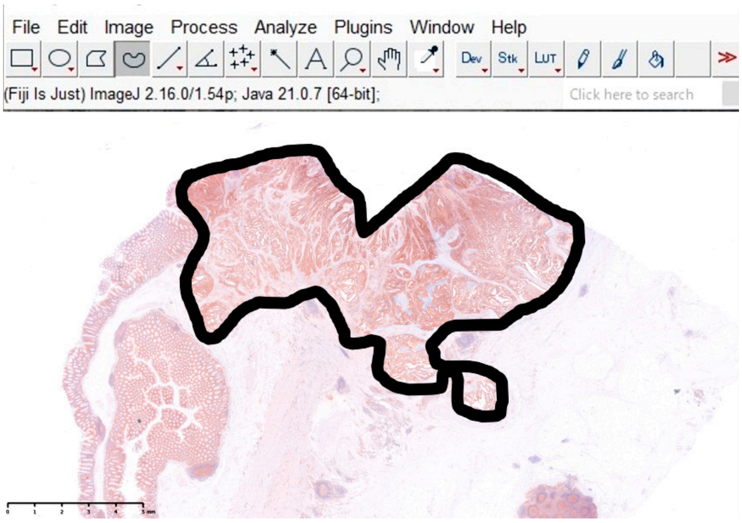


Figure SM14. Manual annotation of tumor regions on Wnt3-stained images. Tumor regions were manually outlined using the Freehand Selection tool. Non-tumorous surrounding tissue was excluded by setting the background color to white (“Edit → Options → Colors”) and clearing areas outside the defined region of interest (“Edit → Clear Outside”). Annotation was performed by one investigator (S.C.) and independently reviewed by another investigator (H.M.).

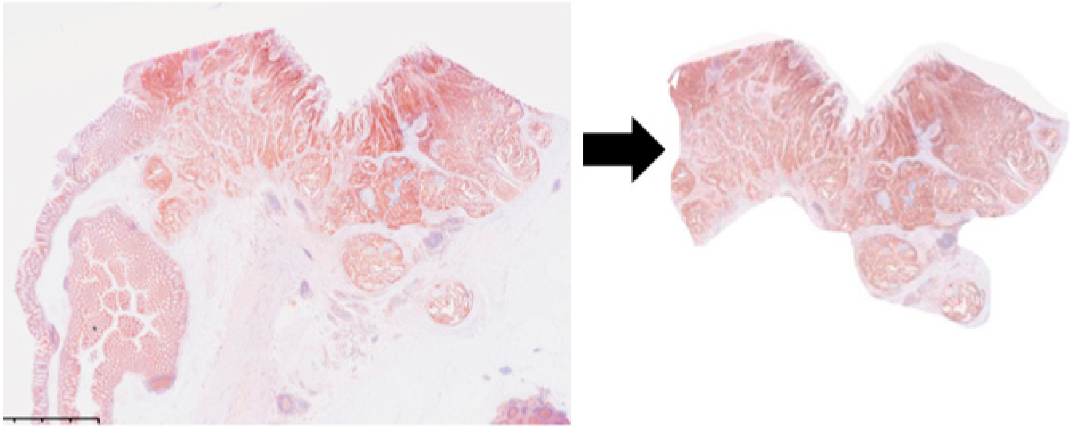

Figure SM15. Exclusion of non-tumorous surrounding tissue.

Non-tumorous surrounding tissue was excluded by setting the background color to white and clearing areas outside the defined tumor region of interest using the “Edit → Clear Outside” function.

*Spatial calibration*

This step was performed by tracing a straight line along the reference scale bar using the *Straight Line* tool, followed by Analyze > Set Scale (red line).

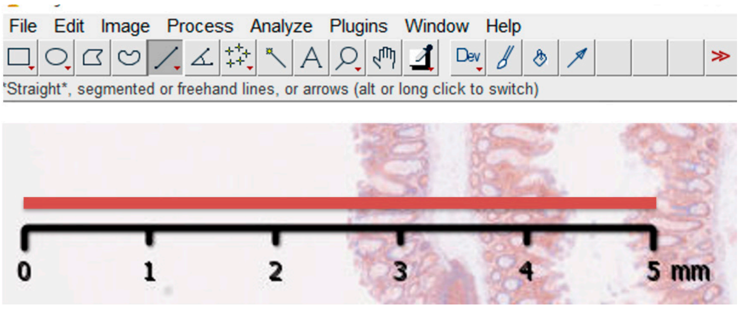


Figure SM16. Spatial calibration using the reference scale bar.
Spatial calibration was performed by tracing a straight line along the reference scale bar and entering the known distance to standardize measurements prior to quantitative analysis.

The known distance (5) and unit of length (mm) were entered, and the scale was applied uniformly across all images.

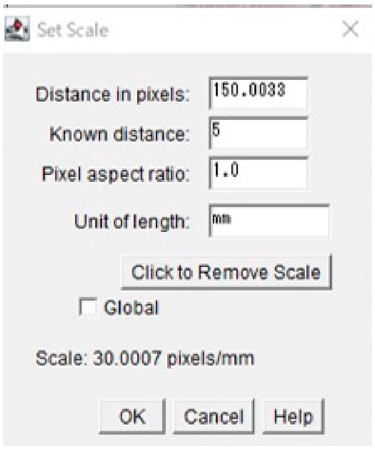

Figure SM17. Entry of the known distance and unit of length. Screenshot showing entry of the known distance and unit of length in the “Set Scale” dialog box to apply spatial calibration uniformly across all images.

*Image preprocessing and thresholding*

Calibrated images were converted to 8-bit grayscale (Image → Type → 8-bit).

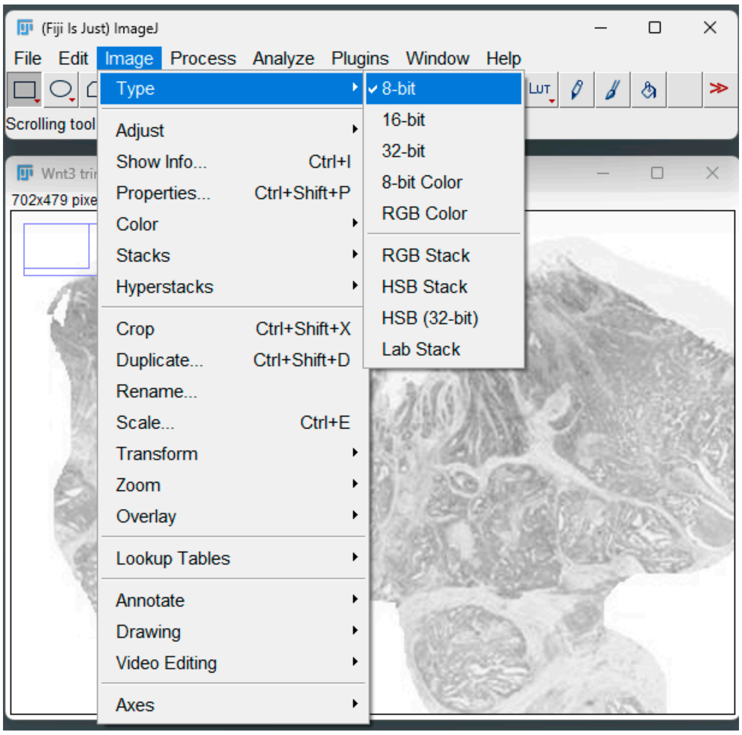


Figure SM18. Conversion of calibrated images to 8-bit grayscale. Calibrated images were converted to 8-bit grayscale format in ImageJ/Fiji prior to threshold-based segmentation to ensure consistent signal intensity processing across cases.

Thresholding was applied to segment Wnt3-positive tumor epithelium from the background. The lower threshold was fixed at 0 (*red box*) to eliminate background noise, whereas the tumor-specific upper threshold values were set to 175 and 200 (*blue boxes*), as determined in the preliminary study of Initial thresholding

determination (n=10). Segmentation was based on Wnt3 staining intensity and morphological features, and identical threshold settings were applied to all cases.

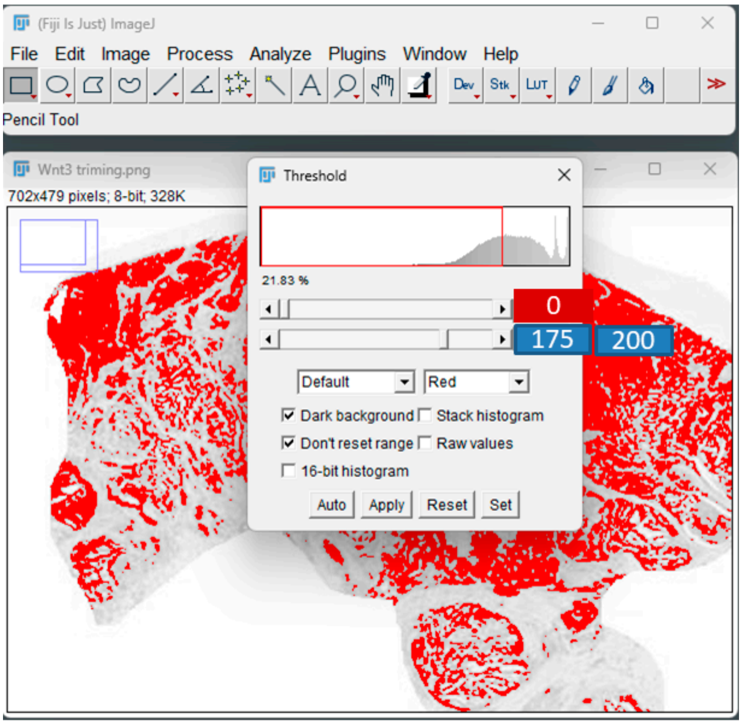


Figure SM19. Threshold settings for Wnt3 segmentation.
Thresholding was applied to segment Wnt3-positive tumor epithelium, with the lower threshold fixed at 0 and upper thresholds set to 175 and 200 as determined in the preliminary study. Identical threshold values were applied uniformly across all cases.

*Area quantification*

Application of these thresholds generated binary images in which immunoreactive tumor epithelium appeared as white particles, whereas surrounding stroma was represented as black. Tumor areas (white pixels) were quantified using Analyze → Analyze Particles with the *Summarize* option enabled. Segmentation accuracy was visually confirmed using the *Overlay* output, and the total area reported in the

Summary table was used as the quantitative measure of immunoreactive tumor epithelium.

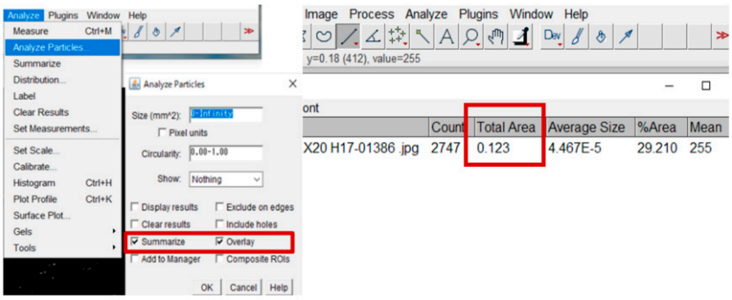


Figure SM20. Binary image generation and particle analysis.

Binary images were generated after thresholding, and immunoreactive tumor areas were quantified using the “Analyze Particles” function with the Summarize option.

*Calculation*

The Wnt3 index (%) was defined as follows:

$$\text{Wnt3 index (\%)} = \frac{\text{immunoreactive tumor area at threshold 175 [mm}^2\text{]}}{\text{immunoreactive tumor area at threshold 200 [mm}^2\text{]}} \times 100$$

*Quality control*

The upper threshold values were determined by consensus between two investigators (S.C. and H.M., the latter being a board-certified pathologist). All image analyses were performed using identical parameter settings to ensure consistency and reproducibility.

## 4. β-catenin index quantification

*Software*

ImageJ/Fiji was used for quantitative analysis of β-catenin (β-CTN) immunohistochemistry.

*Image acquisition*

Three representative tumor fields were captured at ×20 objective magnification as JPEG images from WSIs of β-CTN–stained sections. Capture fields were preferentially selected from the tumor invasive front, where nuclear β-CTN accumulation was most prominent.

*Image analysis procedure*

Each JPEG image was opened in ImageJ/Fiji, following the same preprocessing steps as those described for Wnt3 analysis, except that the known distance (0.1) mm was entered for spatial calibration and the images were converted to 8-bit grayscale.

Thresholding was applied to segment β-CTN–positive tumor epithelium from the background, following the same procedure as in Wnt3 analysis. The lower threshold was fixed at 0 to remove background noise, and upper thresholds were based on the preliminary study of Initial thresholding determination (n=10) by consensus between two investigators (S.C. and H.M.) Upper threshold values (blue boxes) were defined as follows:
(i) 60, to exclude nonspecific staining and detect areas with higher intensity than non-neoplastic mucosa; and
(ii) 145, to approximate overall tumor-rich areas while minimizing stromal staining.
Segmentation was based on β-CTN staining intensity and morphological features. These threshold values were applied uniformly across all analyses.

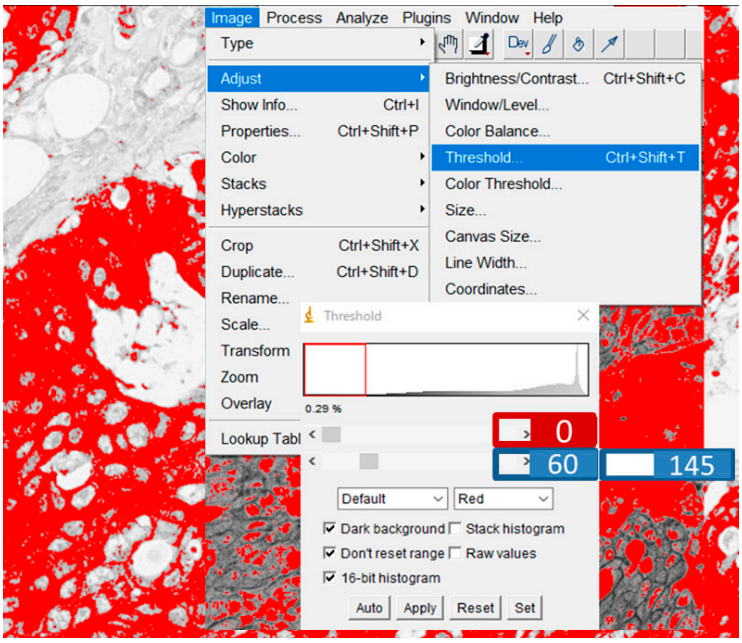


Figure SM21. Threshold settings for β-CTN segmentation.
Thresholding was applied to segment β-CTN–positive tumor epithelium, with the lower threshold fixed at 0 and upper thresholds set to 60 and 145. Identical threshold values were applied uniformly across all cases

Application of these thresholds generated binary images in which immunoreactive tumor epithelium appeared as white particles, whereas surrounding stroma was represented as black. Tumor areas (white pixels) were quantified using Analyze → Analyze Particles with the Summarize option enabled. Segmentation accuracy was visually confirmed using the *Overlay* output, and the total area reported in the *Summary* table was used as the quantitative measure of immunoreactive tumor epithelium.

***Calculation***

The whole β-CTN index (%) was defined as follows:

$$\text{Whole } \beta\text{-CTN index (\%)} = \frac{\text{immunoreactive tumor area at threshold 60 [mm}^2]}{\text{immunoreactive tumor area at threshold 145 [mm}^2]} \times 100$$

Separately, immunoreactive tumor nuclei were manually counted using the Cell Counter plugin in ImageJ/Fiji. Nuclear counts were summed across the same three JPEG images used for whole β-CTN index analysis.

The nuclear β-CTN index (/cm$^2$) was calculated as follows:

$$\text{Nuclear } \beta\text{-CTN index (/cm}^2) = \frac{\text{total number of immunoreactive nuclei}}{\text{immunoreactive tumor area at threshold 145 [cm}^2]}$$

***Quality control***

The upper threshold values were determined by consensus between two investigators (S.C. and H.M., the latter being a board-certified pathologist). All image analyses were performed using identical parameter settings to ensure consistency and reproducibility.
